# The leucine-rich repeats in allelic barley MLA immune receptors define specificity towards sequence-unrelated powdery mildew avirulence effectors with a predicted common RNase-like fold

**DOI:** 10.1371/journal.ppat.1009223

**Published:** 2021-02-03

**Authors:** Saskia Bauer, Dongli Yu, Aaron W. Lawson, Isabel M. L. Saur, Lamprinos Frantzeskakis, Barbara Kracher, Elke Logemann, Jijie Chai, Takaki Maekawa, Paul Schulze-Lefert

**Affiliations:** 1 Department of Plant Microbe Interactions, Max Planck Institute for Plant Breeding Research, Cologne, Germany; 2 Institute of Biochemistry, University of Cologne at Max Planck Institute for Plant Breeding Research, Cologne, Germany; 3 DOE Joint Genome Institute, Berkeley, California, United States of America; 4 Cluster of Excellence on Plant Sciences, Düsseldorf, Germany; University of Queensland, AUSTRALIA

## Abstract

Nucleotide-binding domain leucine-rich repeat-containing receptors (NLRs) in plants can detect avirulence (AVR) effectors of pathogenic microbes. The *Mildew locus a* (*Mla*) NLR gene has been shown to confer resistance against diverse fungal pathogens in cereal crops. In barley, *Mla* has undergone allelic diversification in the host population and confers isolate-specific immunity against the powdery mildew-causing fungal pathogen *Blumeria graminis* forma specialis *hordei* (*Bgh*). We previously isolated the *Bgh* effectors AVR_A1_, AVR_A7_, AVR_A9_, AVR_A13_, and allelic AVR_A10_/AVR_A22_, which are recognized by matching MLA1, MLA7, MLA9, MLA13, MLA10 and MLA22, respectively. Here, we extend our knowledge of the *Bgh* effector repertoire by isolating the AVR_A6_ effector, which belongs to the family of catalytically inactive RNase-Like Proteins expressed in Haustoria (RALPHs). Using structural prediction, we also identified RNase-like folds in AVR_A1_, AVR_A7_, AVR_A10_/AVR_A22_, and AVR_A13_, suggesting that allelic MLA recognition specificities could detect structurally related avirulence effectors. To better understand the mechanism underlying the recognition of effectors by MLAs, we deployed chimeric MLA1 and MLA6, as well as chimeric MLA10 and MLA22 receptors in plant co-expression assays, which showed that the recognition specificity for AVR_A1_ and AVR_A6_ as well as allelic AVR_A10_ and AVR_A22_ is largely determined by the receptors’ C-terminal leucine-rich repeats (LRRs). The design of avirulence effector hybrids allowed us to identify four specific AVR_A10_ and five specific AVR_A22_ aa residues that are necessary to confer MLA10- and MLA22-specific recognition, respectively. This suggests that the MLA LRR mediates isolate-specific recognition of structurally related AVR_A_ effectors. Thus, functional diversification of multi-allelic MLA receptors may be driven by a common structural effector scaffold, which could be facilitated by proliferation of the RALPH effector family in the pathogen genome.

## Author summary

Barley powdery mildew caused by the fungus *Blumeria graminis* forma specialis *hordei* (*Bgh*) can result in annual yield losses of 15% of this cereal crop. *Bgh* promotes virulence in plants through the secretion of diverse effector molecules, small proteins of which a subset enters into and modifies the immune status and physiology of the host leaf. In response, the host has evolved a multitude of disease resistance genes. The *Mildew locus a* (*Mla*) resistance gene stands out because diversification in the host population has generated numerous *Mla* variants encoding multi-domain receptors, each of which can directly recognize an isolate-specific *Bgh* effector, designated as avirulence (AVR_A_) effectors. Recognition of AVR_A_ effectors by MLA triggers plant immune responses, a phenomenon known as isolate-specific resistance, which invariably results in localized host cell death. Here, we identify the powdery mildew effector AVR_A6_ and validate its specific interaction with its matching receptor MLA6. Furthermore, through the use of hybrid receptors constructed from MLA1 and MLA6 as well as MLA10 and MLA22 receptors, we provide insights into the specific domains and amino acid residues generally important for AVR_A_ recognition by MLA receptors. We find that sequence variation in the leucine-rich repeats (LRRs) of multi-allelic MLA receptors determines specific recognition of AVR_A_ effectors. These effectors are sequence-unrelated, but our analysis indicates that they may be structurally related. This data may assist in the future generation of synthetic immune receptors with pre-defined recognition specificities.

## Introduction

Plants have evolved sophisticated innate immune systems to protect themselves against colonization by pathogenic microbes [1,2]. At the population level, a host-adapted pathogenic species is comprised of numerous isolates/races with distinctive genetic blueprints which determine their infection phenotypes on individual accessions (genotypes) of a plant host population. In isolate-specific resistance, individual host accessions often mount a hypersensitive immune response against a subset of pathogenic isolates [3,4]. Isolate-specific resistance is mediated by genetic interactions between plant host resistance (*R*) genes and matching pathogen avirulence (*AVR*) effector genes (gene-for-gene model) [5]. Plant *R* genes often encode intracellular nucleotide-binding domain leucine-rich repeat-containing receptors (NLRs) [6]. These receptors have a characteristic modular domain architecture, consisting of a variable N-terminal Coiled-Coil (CC), Toll-Interleukin (TIR) domain or HeLo domain (named after the fungal HET-S and LOPB proteins), a central NB-ARC (*nucleotide-binding* adaptor shared by APAF-1, certain *R* gene products, and CED-4) domain, and C-terminal leucine-rich repeats (LRRs) [7,8]. The LRRs often constitute a determinant for specific pathogen recognition. NLRs can detect AVRs by direct interaction [9–11], a receptor-integrated decoy [12], or indirectly detecting effector-mediated alterations of a host target [13]. Upon AVR recognition by NLRs, a localized host cell death is typically, but not invariably, associated with receptor-mediated immunity.

Here, we study the *NLR* gene *Mildew locus a* (*Mla*), which has the capacity to confer isolate-specific resistance against both biotrophic basidiomycete and ascomycete fungal pathogens in closely related host cereal species, including wheat and barley. The barley *Mla* locus contains a cluster of *NLR* genes and is orthologous to the *Stem Rust* (*Sr*) resistance loci *Sr33* and *Sr50* in wheat, which confer immunity against specific isolates of the barley powdery mildew *Blumeria graminis* f. sp. *hordei* (*Bgh*) and against the wheat rust pathogen *Puccinia graminis* f. sp. *tritici* (*Pgt*), respectively [14–16]. *Bgh* and *Pgt* are filamentous eukaryotic pathogens that belong to different phyla and diverged from each other approximately 500–650 million years ago [17]. Furthermore, *Resistance to Magnaporthe oryzae* (*RMo1*) confers immunity to the rice blast pathogen in barley, and also maps to the barley *Mla* locus [18].

In barley, the *Mla* gene has undergone tremendous diversification into over 30 different allelic resistance specificities in the host population [19,20]. This is the result of a co-evolutionary arms race in which each *Mla* allele recognizes a matching AVR_A_ effector encoded by a subset of *Bgh* isolates [14]. Prior to the molecular isolation of *Bgh* AVR_A_ effectors, domain swap experiments with MLA1 and MLA6 suggested that the LRR is a determinant of isolate-specific disease resistance, an idea which is further supported by the observation that most sites under positive selection map to the predicted solvent-exposed sites of the LRR [19,20]. A multi-allelic *Powdery mildew 3* (*Pm3*) resistance locus also evolved in wheat populations, in which it confers isolate-specific resistance against the wheat powdery mildew *Blumeria graminis* f. sp. *tritici* (*Bgt*) through recognition of sequence-unrelated but possibly structurally related *Bgt* AVRPM3 effectors [21,22]. Although barley *Mla* and wheat *Pm3* both encode CC-type NLRs, the receptors are sequence-unrelated and map to non-syntenic locations in the genomes of the sister host species. Most sites that are polymorphic between different *Pm3* resistance alleles localize to the LRR [23–26]. For other multi-allelic NLRs, yeast two-hybrid and co-immunoprecipitation experiments with matching effectors suggested that the LRRs determine isolate-specific resistance by direct effector binding [10,27]. However, it remains unclear whether AVRPM3 effectors directly bind to PM3 receptors and whether the LRRs of allelic variants of MLA or PM3 receptors are directly responsible for specific discrimination between powdery mildew avirulence effectors, and thereby for isolate-specific recognition.

Recently, we identified the sequence-unrelated *Bgh* effectors AVR_A1_, AVR_A7_, AVR_A9_, AVR_A13_, and allelic AVR_A10_/AVR_A22_ [11,28], which are recognized by barley MLA1, MLA7, MLA9, MLA13, MLA10 and MLA22, respectively [11]. Experiments in yeast, in the absence of other plant proteins, provided evidence for direct interaction of three receptor-effector pairs, namely MLA7/AVR_A7_, MLA10/AVR_A10_, and MLA13/AVR_A13_ [11]. However, it is not known how MLA receptors with >90% sequence identity can recognize the sequence-unrelated fungal *Bgh* effectors. Structural relatedness between effectors, which is needed for recognition by allelic variants of MLA, could explain this phenomenon. For instance, based on structural predictions, ~15% of candidate-secreted effector proteins (CSEPs) were predicted as RNase-Like Proteins expressed in Haustoria (RALPHs), among them, CSEP0064 [29–31]. The X-ray structure of *Bgh* CSEP0064 indeed revealed a ribonuclease-like fold. Structural overlay of CSEP0064 and the active fungal F1 RNase from *Fusarium moniliforme* demonstrated the absence of canonical catalytic residues in the predicted substrate-binding pocket of CSEP0064 [30]. Notably, AVR_A7_ and AVR_A13_ but not the other isolated *Bgh* AVR_A_ proteins were predicted by IntFOLD version 3.0 to also adopt a RNase-like fold. Here, we used a transcriptome-wide association study (TWAS) approach [11,28] to identify the effector recognized by the barley *Mla6* receptor. The protein which we identified as AVR_A6_, CSEP0254, is very likely structurally similar to the RALPH effector CSEP0064 [30]. By applying version 5.0 of the structural prediction algorithm IntFOLD [32], we found that all identified AVR_A_ effectors are predicted to share structural similarity with fungal RNases, but similar to all other *Bgh* ribonuclease-likes CSEPs, the isolated AVR_A_ effectors also lack the residues critical for catalytic activity [30,31]. By taking advantage of previously engineered hybrid receptors of MLA1 and MLA6 we confirm that the molecular basis of isolate-specific disease resistance against *Bgh* isolates A6 and K1 lies in the specific recognition of AVR_A6_ and AVR_A1_ effectors by six and 12 C-terminal LRRs of MLA1 and MLA6 receptors, respectively. We find that the LRRs of allelic MLA10 and MLA22 are largely sufficient for specific perception of allelic AVR_A10_ and AVR_A22_ effector proteins. Co-expression of hybrid effectors generated from allelic AVR_A10_ and AVR_A22_ with MLA10 and MLA22 receptors revealed that multiple effector residues participate in AVR_A_ recognition specificities. Our findings imply a model in which co-evolution of the barley *Mla*-*Bgh AVR*_*a*_ pathogen interaction is driven MLA sequence diversification upon detection of a common structural effector scaffold. This co-evolutionary process may have contributed to the proliferation and sequence diversification of RALPH effectors in the powdery mildew genome.

## Results

### TWAS identifies *BLGH_00709* (*CSEP0254*) as an *AVR*_*a6*_ candidate

**Fig 1:**
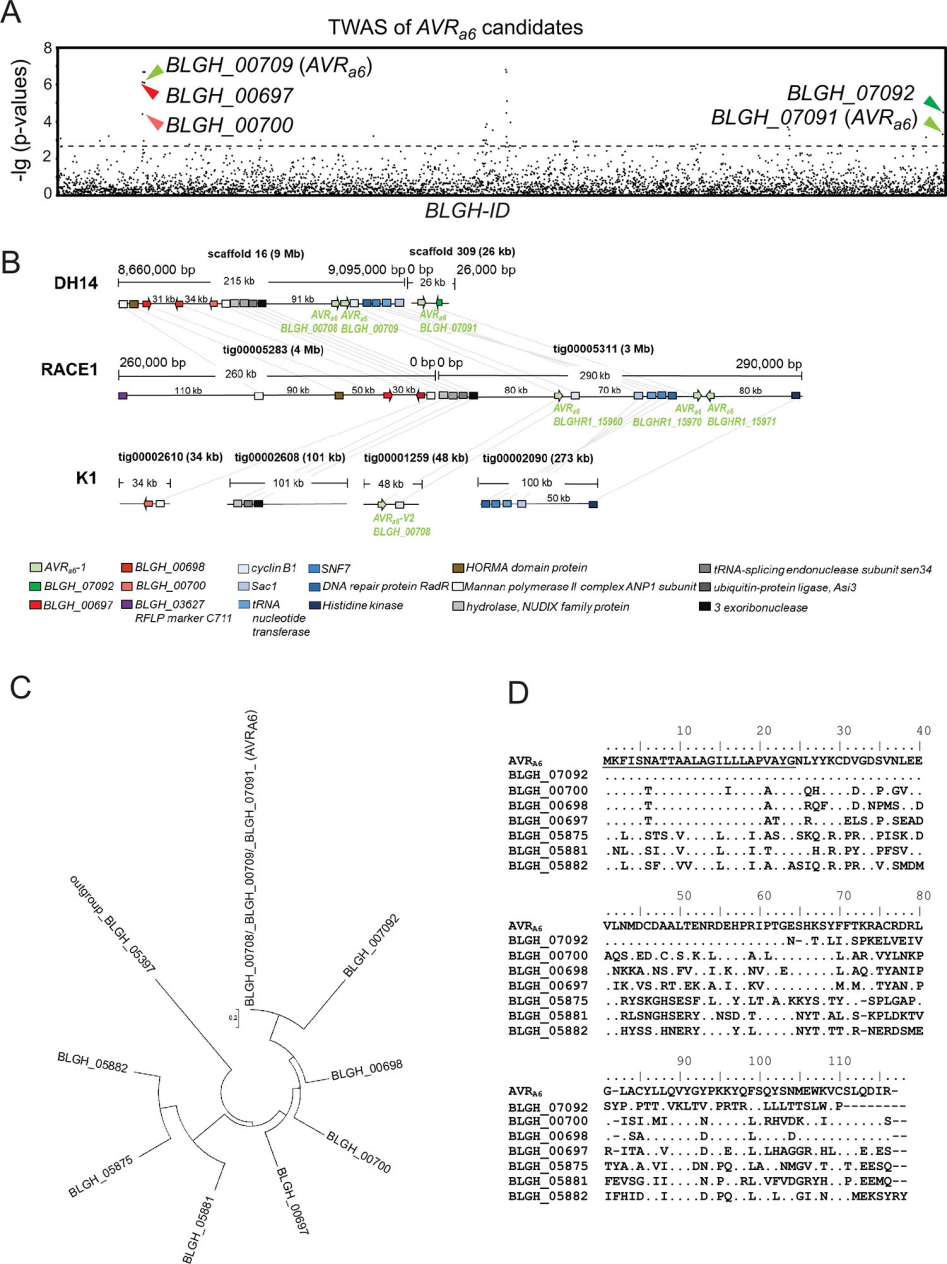
Identification of *BLGH_00709* (*CSEP0254*) as one of the top ranking *AVR*_*a6*_ candidates by association of *Bgh AVR*_*a*_ profiles on *Mla6* near-isogenic lines with transcript polymorphisms. (A) Manhattan plot summarizing the gene-wise association results for candidate *AVR*_*a6*_. The x axis represents the *Bgh* DH14 genes, sorted by *Bgh* gene ID; the y axis shows −lg of p-values for all genes with at least one nonsynonymous coding SNP, indels as well as presence or absence of transcripts. CSEPs with a *p* < 0.018 (dotted line) are depicted by arrowheads. The candidate *AVR*_*a6*_ gene copies *BLGH_00709* (*CSEP0254*) and *BLGH_07091* are designated in the plot with bright green arrowheads. *BLGH_07092* is designated by a dark green arrowhead. The other candidates *BLGH_00697* (*CSEP0058*) and *BLGH_00700* are depicted with a dark red and a bright red arrowhead, respectively. (B) Schematic illustration of the chromosomal regions harboring the *AVR*_*a6*_ candidate *BLGH_00709* and its paralogues and family members with corresponding gene IDs in the genomes of *Bgh* isolates DH14, RACE1, and K1. All CSEPs are depicted by arrows. (C) Phylogeny of CSEP family 8 containing AVR_A6_, which can be divided into clade 1 (BLGH_00709, BLGH_07092, BLGH_00700, BLGH_00698, BLGH_00697) and clade 2 (BLGH_05882, BLGH_05875, BLGH_05881), based on the protein sequences excluding the signal peptide and using BLGH_05397 as an outgroup. (D) Protein sequence alignment of AVR_A6_ and CSEP family 8 members including their respective signal peptides.

For the isolation of *AVR*_*a6*_, we examined the gene-wise association of *Bgh* transcriptomes with the published infection phenotypes of 27 *Bgh* isolates on *Mla6* barley lines [11,28]. We used the previously described *in planta* fungal transcripts of the 27 *Bgh* isolates and their published infection phenotypes on barley *Mla6* near-isogenic lines (NILs) of the barley cultivars (*cv*.) Pallas and Manchuria [11]. In short, we integrated high-confidence non-synonymous variants over each annotated *Bgh* gene and considered absence of a transcript as a missing genotype to obtain gene-wise genotypes. We then tested the gene-wise genotypes for association with the observed avirulence phenotypes using Fisher’s exact test [11]. Association mapping identified a number of genes encoding CSEPs. The *csep* encoding genes with the lowest *p*-values in this analysis were *BLGH_00709* (*CSEP0254*; gene-wise: *p* = 7,40E-07) and *BLGH_00697* (*p* = 7.25E-07), suggesting them to be top-ranking candidates for *AVR*_*a6*_ (Fig 1A, S1 and S2 Tables). To first determine which candidate is recognized by MLA6 in barley, we transiently co-expressed the *AVR*_*a*_ candidate gene and *Mla6* receptor gene in *cv*. Golden Promise barley protoplasts. Co-expression of matching *AVR*_*a*_-*Mla* pairs in this system triggers a reduction in luciferase (LUC) reporter gene activity as a proxy for cell death [33]. Co-expression of *BLGH_00709* and *Mla6* led to a significant reduction of relative LUC activity in comparison to the empty vector (EV) control (98% reduction), while co-expression of *BLGH_00697* with *Mla6* did not reduce LUC activity (S1 Fig). This suggests that *BLGH_00709* is recognized by *Mla6* in barley, and we therefore subjected *BLGH_00709* to further analysis as the top *AVR*_*a6*_ candidate.

First, we analyzed the gene architecture of the *AVR*_*a6*_ candidate gene in the available genomes of the *Bgh* isolates DH14 and RACE1 (both avirulent on *Mla6* lines). The annotated near chromosome-level reference genome of the DH14 *Bgh* isolate harbors two more identical copies of *BLGH_00709*. These copies are annotated as *BLGH_00708* and *BLGH_07091* (Fig 1B). Additionally, DH14 harbors another *CSEP0254* paralog, called *BLGH_07092*. In comparison to *BLGH_00709*, *BLGH_07092* carries a frameshift mutation that predicts an altered sequence from aa 64 onwards in the *BLGH_07092* encoded protein (Fig 1D). *BLGH_00708* and *BLGH_00709* are located in close proximity to each other in a head-to-tail orientation next to the cyclin B1 gene on scaffold 16 of the DH14 genome, while *BLGH_07091* resides with *BLGH_07092* on scaffold 309 in a head-to-head orientation. In the genome of the *Mla6* avirulent RACE1 isolate, three identical copies of *BLGH_00709* can be found on tig00005311: *BLGHR1_15960* (syntenic position to *BLGH_00709*) is located next to the cyclin B1 gene and on the same scaffold *BLGHR1_15970* (syntenic position to *BLGH_07091*) and *BLGHR1_15971* (syntenic position to *BLGH_07092*) reside in a head-to-head orientation. Further analysis of genes surrounding the *AVR*_*a6*_ locus in DH14 and RACE1 suggests major genomic rearrangements: a gene cluster containing a tRNA nucleotide transferase and the DNA repair protein RadR is present in inverted orientation in scaffold 16 and tig00005311, and the *BLGH_00700* family member cannot be found on tig0005283 of the RACE1 genome (Fig 1B). For simplicity, from here on we will refer to the three sequence-identical paralogues *BLGH_00709*, *BLGH_00708*, and *BLGH_07091* as *AVR*_*a6*_. AVR_A6_ is part of the CSEP family 8, which contains six additional members and can be subdivided into two clades: AVR_A6_, BLGH_07092, BLGH_00698 (CSEP0333), BLGH_00700, and BLGH_00697 (CSEP0058) belong to clade 1, whereas BLGH_05881 (CSEP0151), BLGH_05875 (CSEP0147), and BLGH_05882 (CSEP0148) belong to clade 2 (Fig 1C). The clade 1 CSEP family 8 members share a 48.3–60.4% sequence identity with AVR_A6_, while the clade 2 family members share a 32.9–39.7% sequence identity with the avirulence effector candidate. As *BLGH_07092* is most likely a non-functional copy of *AVR*_*a6*_, and its expression is lower when compared to *AVR*_*a6*_ in every *Bgh* isolate (S2 Fig), we did not subject it to further analysis.

Analysis of transcriptomic data revealed that all isolates avirulent on the *cv*. Manchuria and *cv*. Pallas *Mla6* NILs express *AVR*_*a6*_, which encodes a 115-aa-long protein with a predicted 24-aa-long signal peptide (SP) (S3 and S4 Figs). *AVR*_*a6*_ possesses one intron, which is spliced out in all transcripts of avirulent isolates. However, we identified transcripts of *AVR*_*a6*_ carrying this intron in all *Bgh* isolates virulent on *Mla6* NILs. The transcription of the *AVR*_*a6*_ intron may be facilitated by two different mechanisms (S4 Fig): The *AVR*_*a6*_ transcript variant expressed in the virulent isolates CC66 and CC148 exhibits a T270C mutation in the intron branch point consensus sequence, suggesting that intron retention may be caused by inefficient or nonexistent U2 spliceosome recognition (S4B Fig). If this is the case, the intron retention leads to a premature stop codon and a truncated protein, which is only 79 aa-long including the signal peptide. This deduced truncated protein variant, which we named AVR_A6_-V1, would also harbor A48S, A75T, and R79S amino acid substitutions. The isolates K1, K2, K3, B103, S15, S16, S22, and S25 are virulent on *Mla6* lines and contain a splice donor site mutation (S3 and S4B Figs) in the transcripts that maps to the *AVR*_*a6*_ gene of the reference genome. Genome analysis of isolate K1 [11] confirmed this splice-site mutation (Fig 1B). The predicted intron retention in the transcript of the *AVR*_*a6*_ variant expressed by the K1, K2, K3, B103, S15, S16, S22, and S25 isolates leads to a premature stop codon as well as to a predicted 79-aa-long truncated protein (S4B Fig). In addition, the encoded protein exhibits two amino acid substitutions when compared to AVR_A6_: A75T and R79I and we named this variant AVR_A6_-V2. Furthermore, the number of transcripts from virulent *Bgh* isolates that map to *AVR*_*a6*_ is approximately four-fold lower than the *AVR*_*a6*_ transcripts in the avirulent DH14 isolate (S2 Fig), which could be a consequence of nonsense-mediated mRNA decay. In conclusion, virulence of *Bgh* isolates on barley NILs harboring *Mla6* is likely conferred by SNPs in splice sites that may lead to intron retention in the respective genes. This is associated with reduced levels of the transcripts that map to the *AVR*_*a6*_ gene in *Bgh* isolates virulent on *Mla6* lines.

### Transient co-expression assays provide evidence for specific recognition of *AVR*_*a6*_ by *Mla6*

**Fig 2:**
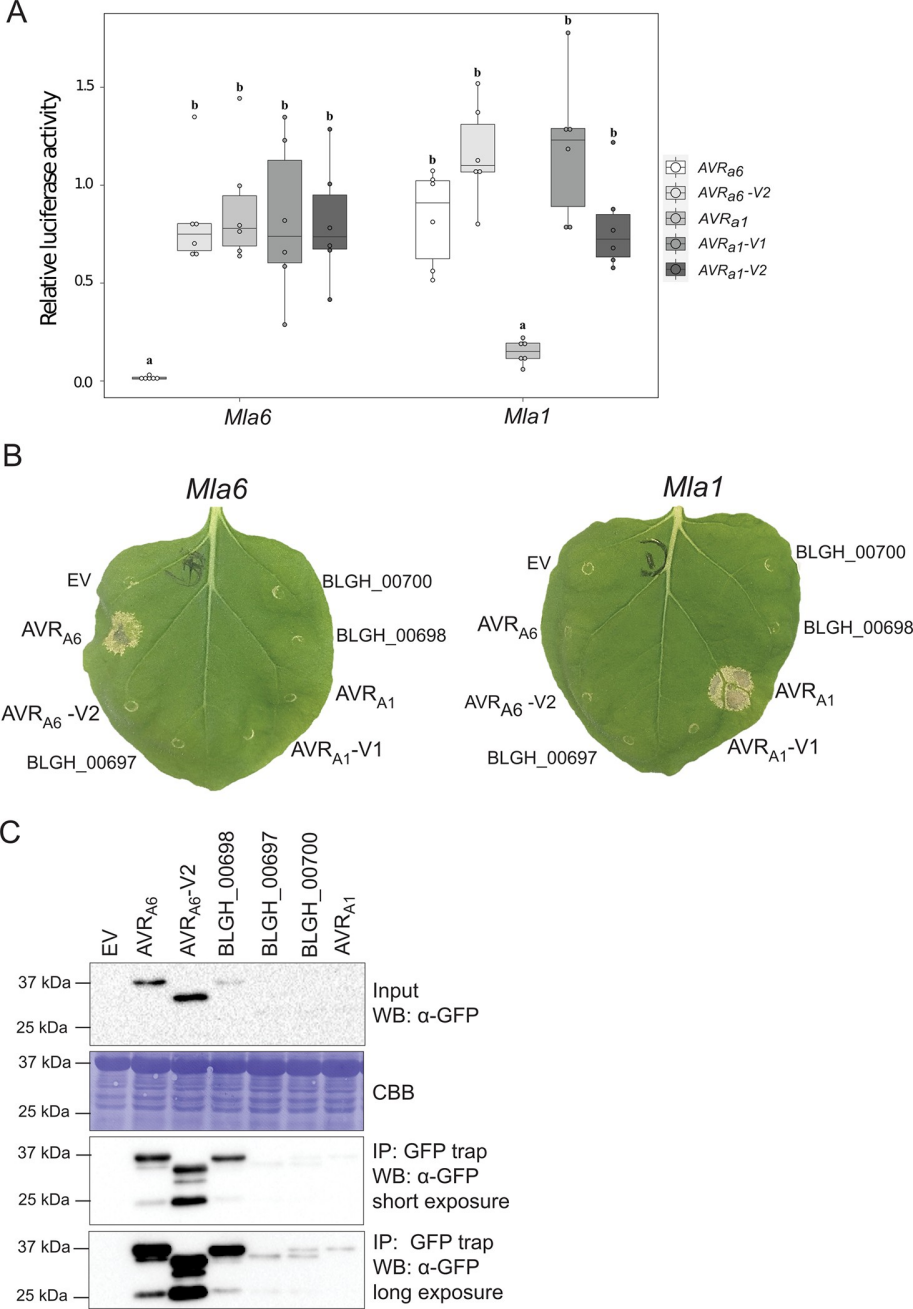
*Mla6* and *AVR*_*a6*_ co-expression in barley protoplasts and *N*. *benthamiana* causes a specific cell death response. (A) Barley *cv*. Golden Promise protoplasts were transfected with pIPKb002 vectors containing cDNAs of *Mla6* or *Mla1* and either an empty vector (EV), *AVR*_*a6*_, *AVR*_*a6*_*-V2*_,_ *AVR*_*a1*,_ *AVR*_*a1-*_*V1*, or *AVR*_*a1*_*-V2* variants lacking their respective signal peptides together with a *pUBI*:*Luciferase* construct. The LUC activity relative to the EV sample was measured as a proxy for cell death 16 h post transfection. Box plot diagrams show median of the relative LUC activity of six independent transfections, which are represented by dots, while the box shows the interquartile range. Significant differences between samples were analyzed using non-parametric Kruskal-Wallis (KW) analysis followed by a Dunn’s test. Calculated KW *p*-values are as follows: *Mla6*: *p* = 0.007146; *Mla1*: *p* = 0.0007392. Samples labeled with identical letters did not differ significantly (*p* < 0.05) in the Dunn’s test for the corresponding *Mla* variant. (B) cDNAs of clade 1 *AVR*_*a6*_ family members *BLGH_00698*, *BLGH_00697*, *BLGH_00700*, *AVR*_*a6*_, and *AVR*_*a1*_ variants were expressed without their respective signal peptides and stop codons and with a C-terminal mYFP fusion under the control of a 35S promotor in *N*. *benthamiana*. The effectors were co-expressed with *Mla1* and *Mla6* cDNAs fused C-terminally with a 4xmyc tag under the control of a 35S promotor. Cell death was scored five days post infiltration and Figures show a representative of at least 15 co-transformations. (C) Protein levels of AVR_A1_-mYFP, AVR_A6_-mYFP, AVR_A6_-V2-mYFP, BLGH_00698-mYFP, BLGH_00697-mYFP and BLGH_00700-mYFP. Samples for total protein extraction were harvested two days post infiltration. mYFP fusion proteins were enriched by an GFP-Trap. Proteins were separated using 10% or 12% polyacrylamide gels and proteins were detected using α-GFP and α-myc western blotting (WB). IP = immunoprecipitation. CBB = Coomassie Brilliant Blue.

To determine if the candidate AVR_A6_ is specifically recognized by MLA6, we first co-expressed *AVR*_*a6*_ and *Mla6* in a transient barley protoplast system containing leaf mesophyll cells [33]. Co-expression of matching *AVR*_*a6*_ or *AVR*_*a1*_ with *Mla6* or *Mla1*, respectively, triggered significant reductions in LUC activity, when compared to reference samples where the effector gene has been exchanged to an EV (98% and 85% reductions, respectively, Fig 2A). Co-expression of *Mla6* with a DNA sequence that encodes a truncated version of *AVR*_*a6*_ (likely encoded by *AVR*_*a6*_*-V2*) or co-expression of *AVR*_*a6*_ with *Mla1* did not lead to significantly reduced LUC activity compared to samples co-expressing *AVR*_*a6*_ with *Mla6*, confirming the specificity of the recognition (Fig 2A).

To examine whether AVR_A6_ is recognized by MLA6 in a heterologous expression system without the presence of other barley proteins, we co-expressed the C-terminally mYFP-tagged effector fusion protein with the C-terminally 4xmyc-tagged receptor in *N*. *benthamiana*. Unlike the essentially complete cell death observed in the barley protoplast system (as evidenced by the very low levels of LUC activity), co-expression of *AVR*_*a6*_*-mYFP* and *Mla6-4xmyc* triggered a cell death of varying confluence in *Agrobacterium tumefaciens* infiltrated tissue in independent *N*. *benthamiana* leaves compared to the cell death observed when co-expressing *AVR*_*a1*_ and *Mla1* (Figs 2B and S14). Co-expression of *AVR*_*a6*_*-mYFP* with *Mla1*-*4xmyc* did not elicit cell death, confirming the specific recognition of candidate AVR_A6_ by MLA6 but not MLA1. No cell death was observed when *Mla6-4xmyc* was co-expressed with a DNA sequence that encodes a truncated version of *AVR*_*a6*_*-mYFP* (here named AVR_A6_-V2-mYFP), even though both AVR_A_ and MLA proteins are detectable in *N*. *benthamiana* leaf extracts (Figs 2B, 2C and 5C). Furthermore, co-expression of the clade 1 CSEP family 8 member *BLGH_00698-mYFP*, which shares the highest sequence similarity with *AVR*_*a6*_, with *Mla6* did not lead to cell death, even though BLGH_00698-mYFP is detectable in *N*. *benthamiana* leaf extracts (Fig 2B and 2C). Detection of BLGH_00700 and BLGH_00697 proteins was possible only after enrichment with a GFP-Trap, suggesting that their protein stability is lower than those of AVR_A6_ and BLGH_00698 in this system. A faster-migrating protein band for BLGH_00697-mYFP and a double band visible after blotting for BLGH_00700-mYFP suggest that these proteins may either be cleaved post-translationally by proteases in heterologous *N*. *benthamiana* or that they are not stable in the plant extraction buffer (Fig 2C). Taken together, co-expression of AVR_A6_ with MLA6 in both homologous and heterologous plant expression systems triggers a significant and specific cell death response, indicating specific effector recognition by the matching MLA immune receptor.

### AVR_A_ effectors have low sequence similarity, but show predicted structural homology to RNases

**Fig 3:**
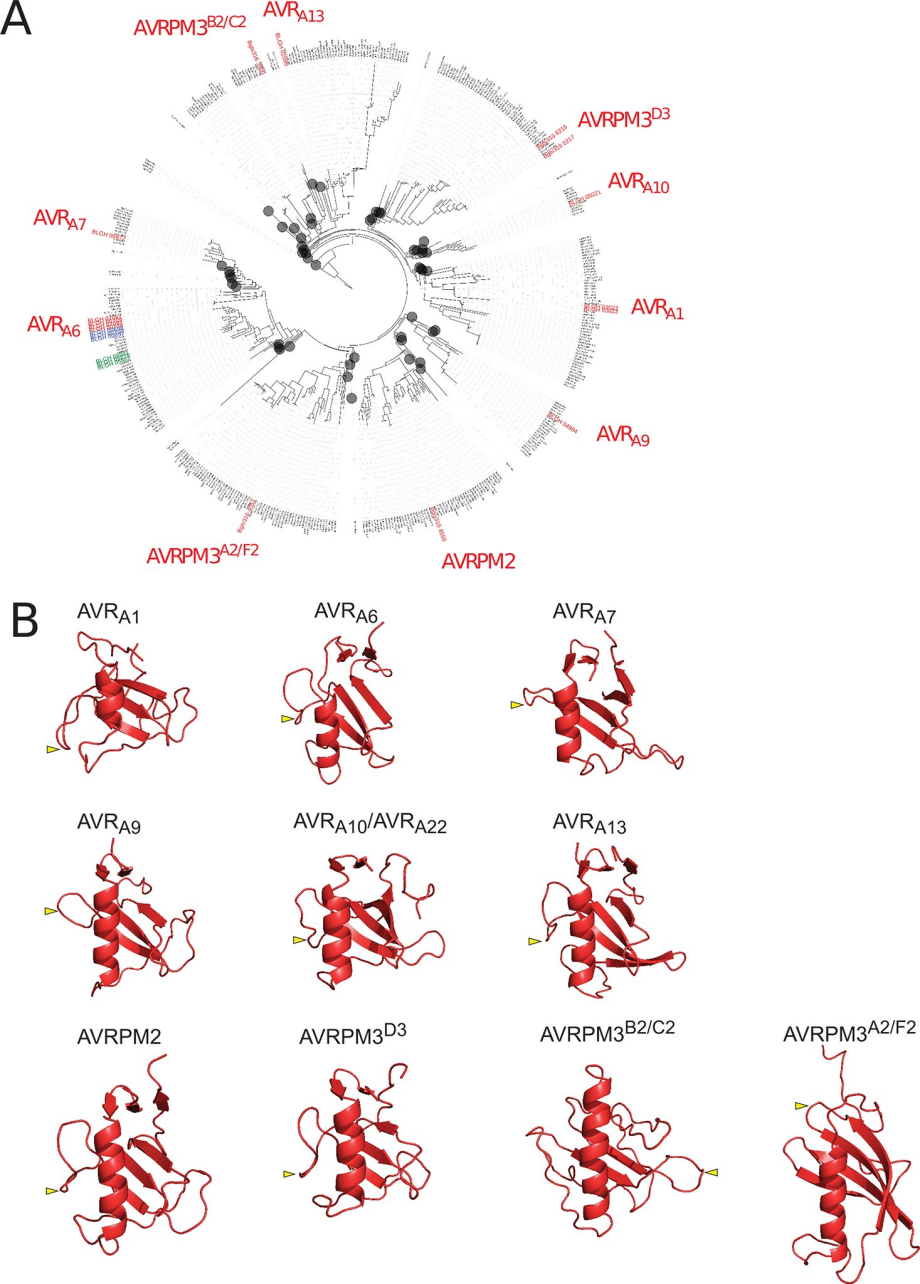
*Bgh* AVR_A_ and *Bgt* AVRPM effectors are sequence-unrelated but exhibit predicted structural similarity to RNases. (A) Maximum likelihood phylogeny including all predicted CSEPs from *B*. *graminis* formae speciales *poae*, *lolium*, *avenae*, *tritici* 96224, *hordei* DH14, *secalis* S1459, *triticale* T1-20, and *dactylidis*. Depicted in red are the BLGH-IDs of all so far isolated *Bgh* AVR_A_ and *Bgt* AVRPM effectors. Depicted in blue are the clade-1 family members of AVR_A6_, while the clade-2 family members are colored in green. CSEP clades that were collapsed (grey circles) to improve legibility of the tree do not include AVR members and are indicated by grey circles. (B) Structural prediction of isolated AVR_A_s and AVRPM by IntFOLD version 5.0 in red (p-values: AVR_A1_ = 4.888e^-4^ most similar to PDB IDs 5gy6, 3who and 1rds, AVR_A6_ = 3.293e^-5^ to 6fmb, AVR_A7_ = 2.114e^-4^ to PDB ID 5gy6, AVR_A9_ = 1.18e^-5^ most similar to PDB IDs 6fmb, 3who, and 1ch0, AVR_A10_ = 9.759e^-5^ most similar to PDB ID 1fusa and to 3whoa, AVR_A13_-1 = 7,359e^-7^ most similar to 6fmb, AVRPM2 = 8.741e^-9^ most similar to 6fmb, 1chOA, and 1rmsA, AVRPM3^D3^ = 8.82e^-5^ most similar to PDB 6fmb and 5gy6A, AVRPM3^A2/F2^ = 1.145e^-5^ to 3ub1A2, AVRPM3^B2/C2^ = 7.079e^-2^, no structural similarities predicted). Yellow arrow depicts relative position of the characteristic RALPH intron in effector structures.

We subjected AVR_A6_ to a phylogenetic analysis including all annotated CSEPs in *B*. *graminis* formae speciales *poae*, *lolium*, *avenae*, *tritici* (isolate 96224), *hordei* DH14, *secalis* (isolate S1459), *triticale* (isolate T1-20), and *dactylidis*, but were unable to detect significant polypeptide sequence relatedness to other known *Bgh* AVR_A_ effector proteins or to the so far isolated wheat powdery mildew avirulence effectors, AVRPM2, AVRPM3^A2/F2^, AVRPM3^B2/C2^, and AVRPM3^D3^ (Fig 3A). However, we noted that all the avirulence proteins isolated from barley and wheat powdery mildews belong to CSEPs with a length of approximately 80 to 130 amino acids when neglecting their respective signal peptides. The same is true for CSEP0064, which was shown to form a RNase-like protein structure. To determine potential structural similarity between AVR_A6_ and known *Bgh* effectors, we subjected AVR_A6_ to structural prediction using IntFOLD version 5.0 [32]. AVR_A6_ exhibited high predicted structural similarity to the RNase-like fold observed for the X-ray structure, suggesting that AVR_A6_ possesses a ribonuclease-like fold similar to AVR_A13_ and AVR_A7_ (S5 Fig). This prompted us to also reanalyze all isolated AVR_A_ effectors using the with IntFOLD version 5.0. We found that AVR_A1_, AVR_A9_, and AVR_A10_/AVR_A22_ are also predicted to harbor a central α-helix directly facing three to four β-sheets with a topology characteristic of ribonucleases (Fig 3B). This is reminiscent of structural predictions for *Bgt* effectors AVRPM2 and AVRPM3^D3^, which also suggested structural similarities to ribonucleases (Fig 3, [22,34–36]). Although the relationship to ribonucleases is less clear for *Bgt* AVRPM3^A2/F2^ and AVRPM3^B2/C2^, these two effectors were predicted to exhibit a central α-helix and two to four ß-sheets like AVRPM3^D3^ ([22], Fig 3B). We examined *AVR*_*a*_ and *AvrPm* gene models for the presence of an intron, which is thought to be characteristic for RALPH effector-encoding genes [29,31] and identified one intron at position +163 to +201 bp after the end of the signal peptide (S6A and S7A Figs). The relative intron position in the structural predictions is in an unstructured loop between the first and the second ß-sheet, except in AVR_A13_, AVRPM3^A2/F2^, and AVRPM3^B2/C2^ (Fig 3B). For the latter three effectors, the intron is positioned in a predicted unstructured loop C-terminal to the second ß-sheet (Figs 3B and S6). No common sequence motifs are detectable in the highly diverse intron sequences (S7B Fig). Thus, it remains unclear whether all known *Blumeria* avirulence effectors are descendants of one common “*ur*-RALPH” ancestor [29]. Irrespective of this, our analysis suggests that isolated *Bgh* and *Bgt* avirulence effectors are structurally related to fungal ribonucleases.

### RNase-like AVR_A_ effectors do not show ribonuclease activity

**Fig 4:**
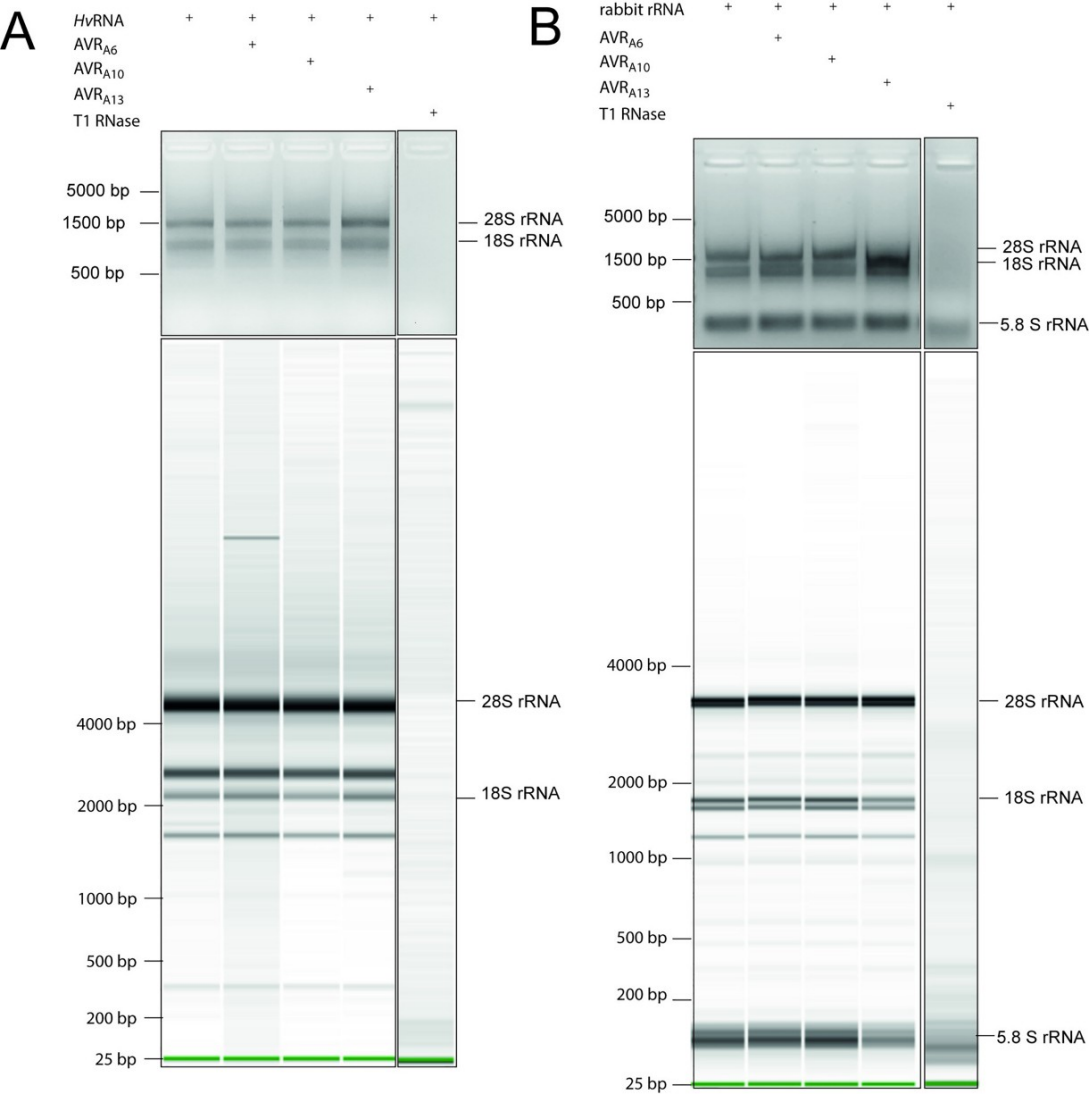
Recombinant AVR_A6_, AVR_A10_, and AVR_A13_ effector proteins do not exhibit ribonuclease activity. To test for ribonuclease activity, heterologous AVR_A6_, AVR_A10_, and AVR_A13_ proteins, purified upon expression of the respective genes in *E*. *coli*, or T1 RNase were co-incubated with (A) denatured *Hv*RNA and (B) native rabbit rRNA always. All samples were separated on non-denaturing 2% agarose gels (top panels) and analysed on a Bioanalyzer (lower panels) to check for RNA degradation.

Using an RNase activity assay, we tested whether AVR_A_ proteins are truly catalytically inactive as suggested for RNase-like *Bgh* effectors previously [29,30]. First, we expressed N-terminally GST-tagged AVR_A6_, AVR_A10_, and AVR_A13_ in *Escherichia coli* and purified them by GST affinity chromatography. We then cleaved the GST tag and applied size-exclusion chromatography (S8A Fig). Successful protein expression and purification of the AVR_A_ effector proteins was tested by SDS-PAGE (S8B Fig). We then incubated AVR_A6_, AVR_A10_, and AVR_A13_ effectors with denatured *Hv*RNA or native rRNA to test for ribonuclease activity [37]. Using RNA gel electrophoresis, we observed a degradation of RNA when co-incubated with a commercially available T1 RNase, which has the same function as the *Fusarium* F1 RNase. We did not observe RNA degradation when incubating *Hv*RNA or rRNA with the AVR_A_ effectors (Fig 4A and 4B). These results were independently validated with AVR_A6_, AVR_A10_, and AVR_A13_ effector proteins that were produced in eukaryotic insect cells, followed by affinity chromatographic purification (S9A and S9B Fig). Taken together, the data indicate that AVR_A_ effectors have no RNase activity, which is consistent with the previous prediction that ascribed pseudoenzymatic function to the RALPHs [29,30].

### The C-terminal leucine-rich repeats of MLA1 and MLA6 receptors account for specific discrimination of structurally homologous AVR_A1_ and AVR_A6_ effectors *in planta*

**Fig 5:**
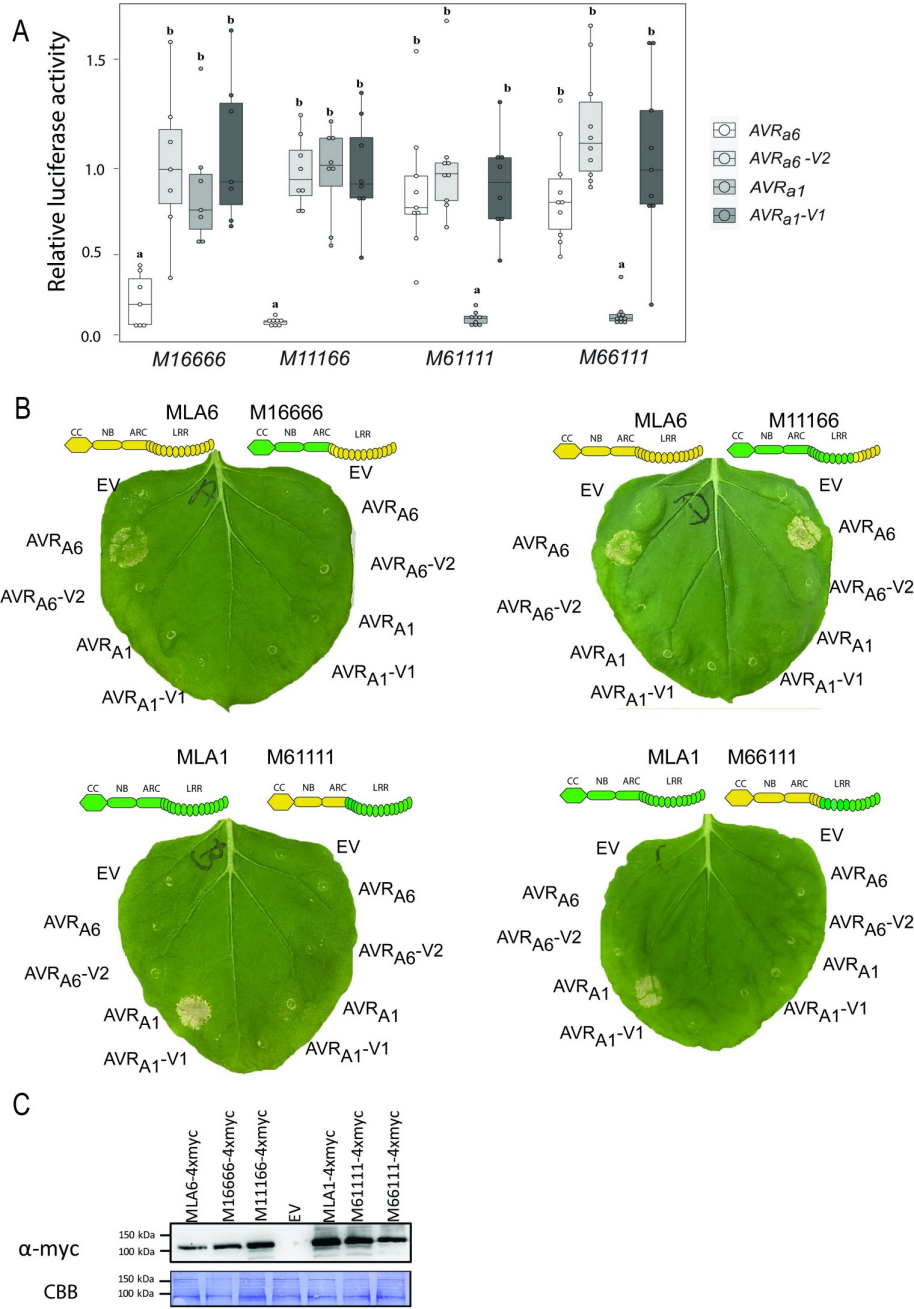
Specific recognition of AVR_A6_ and AVR_A1_ by MLA1/MLA6 chimeric constructs *in planta*. (A) Barley *cv*. Golden Promise protoplasts were transfected with a LUC reporter construct and pIPKb002 vectors containing cDNAs of *AVR*_*a6*_, *AVR*_*a6*_*-V2*, *AVR*_*a1*_, *AVR*_*a1*_*-V1* or an empty vector (EV) together with vectors harboring intron-containing DNA of receptor chimeras *M16666*, *M11166*, *M61111*, or *M66111* under the control of a *pZmUBI* promotor. Transfections were performed at least seven times independently. Significant differences between samples were analyzed using non-parametric Kruskal-Wallis (KW) analysis followed by a Dunn’s test. Calculated KW p-values are as follows: *M16666*: *p* = 0.001883; *M11166*: *p* = 0.000559, *M61111*: *p* = 0.0001582, *M66111*: *p* = 1.658e-05. Samples labeled with identical letters did not differ significantly (*p* < 0.05) in the Dunn’s test for the corresponding *Mla* variant. (B) Transient transformation of *N*. *benthamiana* leaves with empty vector (EV) or cDNAs of *AVR*_*a6*_ or *AVR*_*a1*_ variants fused C-terminally with a mYFP tag together with *Mla1* or *Mla6* cDNAs or *M16666*, *M11166*, *M61111*, or *M66111* intron-containing DNAs with a C-terminal 4xmyc fusion. All constructs were expressed from a 35S promotor. Figures show a representative of at least three independent co-transformations. (C) MLA-4xmyc proteins were extracted two days post infiltration and separated using a 10% polyacrylamide gels and detected using α-myc western blotting, CBB = Coomassie Brilliant Blue.

A previous study showed that most of the residues under positive selection in allelic MLA resistance specificities in barley populations are located in the LRR region [19]. Using single-cell expression of MLA chimeras in barley leaf epidermal cells, C-terminal LRR regions of *Mla1* and *Mla6* were shown to encode determinants for isolate-specific immunity in barley to *Bgh* isolates K1 (carrying *AVR*_*a1*_) and A6 (carrying *AVR*_*a6*_) [38]. Here, we tested if the LRRs determine isolate-specific immunity by specifically recognizing AVR_A_ effectors in barley. Therefore, we made use of the previously constructed intron-containing DNAs of the chimeric receptors *M16666*, *M11166*, *M61111*, and *M66111* ([38] (protein sequence shown in S10 Fig) and co-expressed them with matching *AVR*_*a6*_, *AVR*_*a6*_*-V2*, *AVR*_*a1*_, and *AVR*_*a1*_*-V1* cDNAs lacking the signal peptide (SP) in the pIPKb002 vector under the control of a strong maize ubiquitin promoter in barley protoplasts. Upon co-expression of *AVR*_*a6*_ with *M16666* or *M11166* we detected a significant 79% or 92% reduction in relative LUC activity when compared to the EV samples, respectively (Fig 5A). This reduction was not observed when *M16666* or *M11166* were co-expressed with *AVR*_*a6*_*-V2*, *AVR*_*a1*_, or *AVR*_*a1*_*-V1*, respectively, suggesting that both chimeric receptors specifically recognize their matching effector (Fig 5A). These findings suggest that the last six C-terminal leucine-rich repeats of a total of 15 deduced LRRs in MLA6 account for the recognition specificity of AVR_A6_ in barley. Furthermore, we discovered that co-expression of *AVR*_*a1*_ with *M61111* or *M66111* triggered a significant and specific 92% reduction of relative LUC activity in barley protoplasts indicative of a cell death response compared to the EV sample (Fig 5A). This suggests that, out of 15 predicted LRRs in the MLA1 receptor, the 12 C-terminal ones contribute to the specific recognition of AVR_A1_. Together, these findings corroborate previous experiments that show a significant growth reduction of *Bgh* isolate A6 when barley cells express *M16666* and *M11166*, or growth reduction of *Bgh* isolate K1, when barley cells express *M61111* or *M66111* [38]. While our data does not exclude that other MLA domains contribute to the association with the *Bgh* AVR_A_ effector proteins, we conclude that the MLA LRR regions confer *Bgh* recognition specificities to of the different *Mla* alleles in barley.

To determine if these results can be independently validated in a heterologous system, we co-expressed C-terminally 4xmyc-tagged *M16666*, *M11166*, *M61111*, and *M66111* and C-terminally mYFP-tagged *AVR*_*a6*_ and *AVR*_*a1*_ variants in *N*. *benthamiana*. We observed a strong cell death response upon co-expression of *M11166*-*4xmyc* with *AVR*_*a6*_*-mYFP*, suggesting that the last six C-terminal LRR repeats of MLA6 are sufficient for recognition of *AVR*_*a6*_ even in the absence of further barley-specific host proteins (Fig 5B). We detected weak recognition of AVR_A1_ by M61111 under UV light at 302 nm in *N*. *benthamiana* (indicative of accumulation of autofluorescent compounds in dying plant cells; S11 and S14 Figs), whereas the specific recognition of AVR_A6_ by M16666 and AVR_A1_ by M66111 seen in barley protoplasts was completely lost, suggesting that other barley-specific protein(s) might be necessary for the functionality of these chimeric receptors in the heterologous expression system.

### The LRRs of the MLA10 and MLA22 receptors specifically recognize allelic AVR_A10_ and AVR_A22_

**Fig 6:**
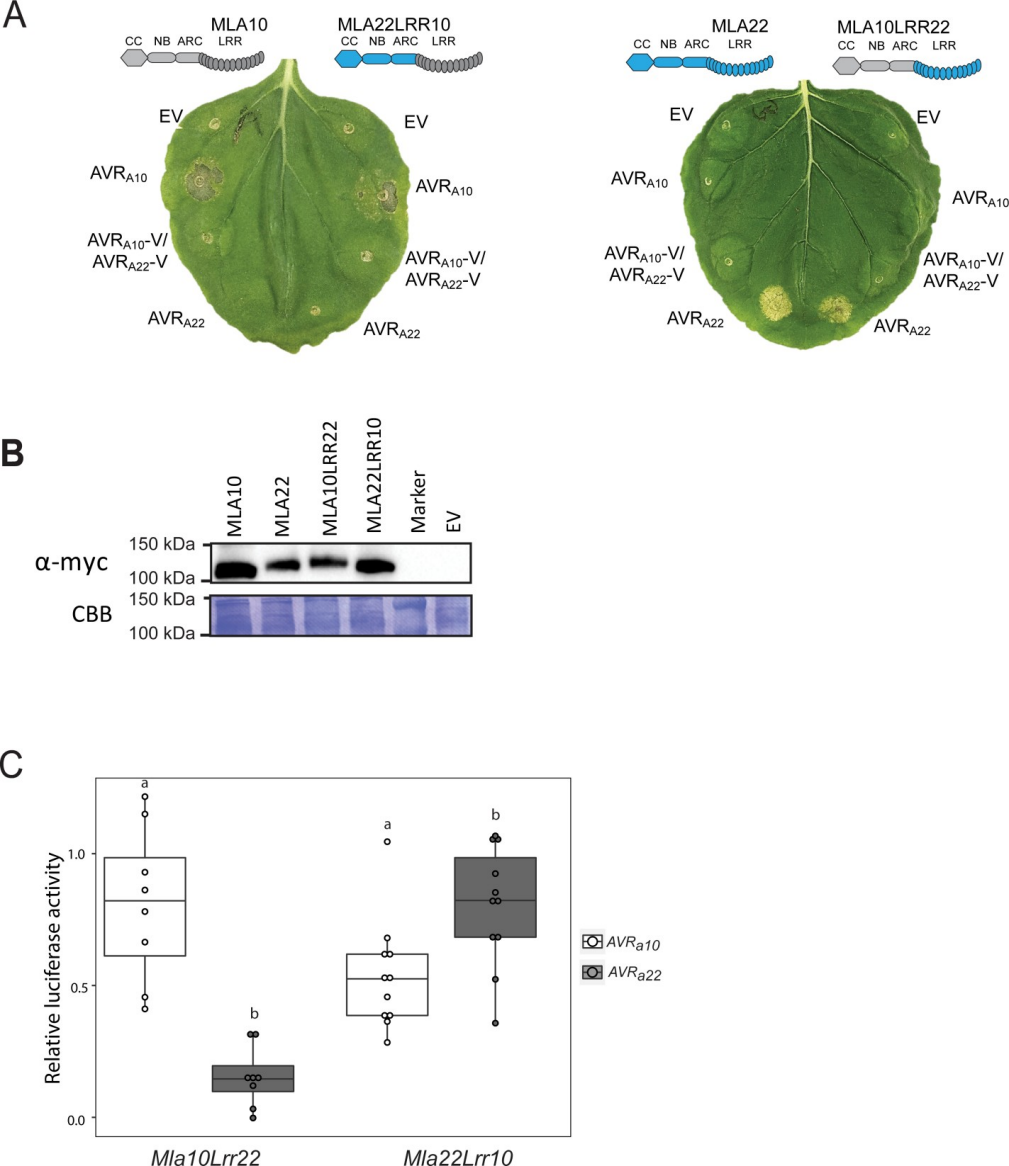
The LRR domains of MLA10 and MLA22 distinguish between AVR_A10_ and AVR_A22_. (A) Transient transformation of *Nicotiana benthamiana* leaves with EV or cDNAs of *AVR*_*a10*,_ *AVR*_*a10*_*-V/AVR*_*a22*_*-V*, and *AVR*_*a22*_ fused C-terminally with mYFP together with *Mla10* or *Mla22* cDNAs fused C-terminally with a 4xmyc tag. All constructs were expressed from a 35S promotor. Cell death was scored five days post infiltration (dpi) and Figures show a representative of at least 15 co-transformations. (B) Protein levels of MLA-4xmyc after total protein extraction from *N*. *benthamiana* leaves at two dpi. Proteins were separated on a 10% polyacrylamide gel and a detected using α-myc western blotting (WB) (C) Barley *cv*. Golden Promise protoplasts were transfected with a LUC reporter construct and pIPKb002 vectors containing cDNAs of *AVR*_*a10*_ or *AVR*_*a22*_ together with *Mla10Lrr22* and *Mla22Lrr10* chimeras. Transfections were performed at least eight times independently. Significant differences between samples were analyzed using non-parametric Kruskal-Wallis (KW) one-way analysis of variance. Calculated KW *p*-values are as follows: Mla10Lrr22: *p* = 0.0007775; Mla22Lrr10: *p* = 0.01654. Samples labeled with different letters differed significantly (*p* < 0.05).

To test if the LRRs of MLA NLRs are necessary to specifically recognize not only sequence unrelated, but also sequence related allelic AVR_A_ effectors, we designed two chimeric receptor genes encoding the MLA10 CC-NB fused with the MLA22 LRRs (MLA10LRR22) and the MLA22 CC-NB domain fused with the MLA10 LRRs (MLA22LRR10) (S12 Fig). Subsequently, *Mla10*-*4xmyc*, *Mla22*-*4xmyc*, *Mla10Lrr22*-*4xmyc* or *Mla22Lrr10*-*4xmyc* were co-expressed with *AVR*_*a10*_*-mYFP*, *AVR*_*a10*_*-V/AVR*_*a22*_*-V-mYFP*, or *AVR*_*a22*_*-mYFP* in heterologous *N*. *benthamiana*. Co-expression of *Mla10-4xmyc* or *Mla22Lrr10-4xmyc* with *AVR*_*a10*_*-mYFP* led to cell death in *N*. *benthamiana* leaves, but no cell death was observed when these *Mla NLRs* were co-expressed with an empty vector (EV), *AVR*_*a10*_*-V / AVR*_*a22*_*-V-mYFP*, or *AVR*_*a22*_*-mYFP* (Fig 6A). Additionally, expression of *Mla22-4xmyc* and *Mla10Lrr22-4xmyc* led to cell death when co-expressed with *AVR*_*a22*_*-mYFP*, but not when these proteins were co-expressed with *AVR*_*a10*_*-mYFP*, *AVR*_*a10*_*-V/AVR*_*a22*_*-V-mYFP*, or an EV control (Fig 6A). The MLA10LRR22 and MLA22LRR10 receptor chimeras were detectable in *N*. *benthamiana* leaf extracts at levels comparable with the MLA10 and MLA22 receptors (Fig 6B). To determine whether these results were reproducible in the homologous barley protoplast system, we expressed *Mla10Lrr22* or *Mla22Lrr10* together with *AVR*_*a10*_ and *AVR*_*a22*_ in leaf protoplasts and measured reduction of LUC activity as a proxy for cell death. In comparison to the EV reference sample, co-expression of *AVR*_*a22*_ with *Mla10Lrr22* and *AVR*_*a10*_ with *Mla22Lrr10* lead to an average 80% and 40% reduction of relative LUC activity, respectively, and this was not the case when *AVR*_*a10*_ was co-expressed with *Mla10Lrr22* or when *AVR*_*a22*_ was co-expressed with *Mla22Lrr10* (Fig 6C). Taken together, the results suggest that the 58 amino acid differences between the LRRs of *Mla10* and *Mla22* are major determinants of respective recognition specificities for the allelic AVR_A10_ and AVR_A22_ effectors.

**Fig 7:**
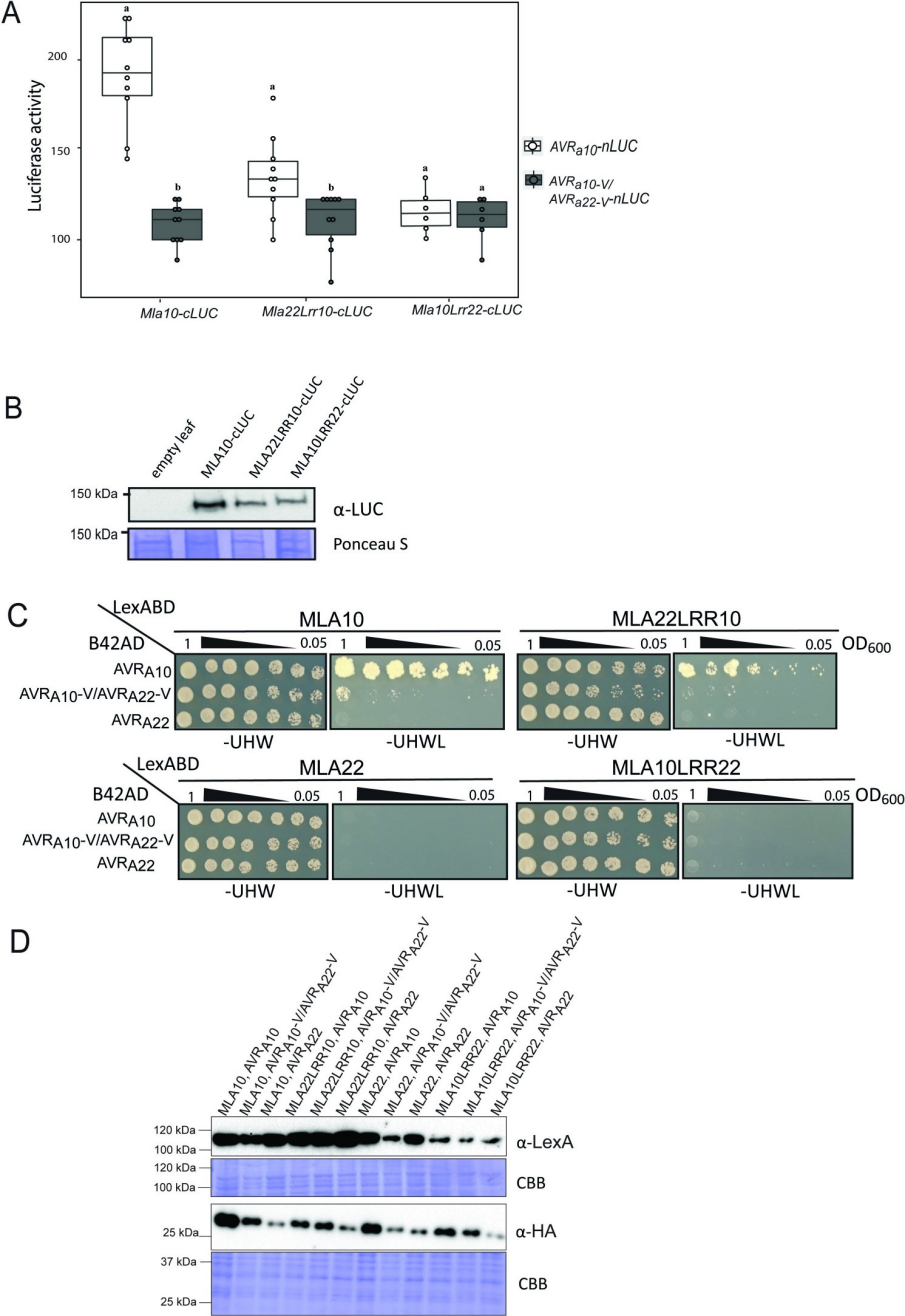
The LRR domain of MLA10 accounts for the specific interaction with AVR_A10_ *in planta* and in yeast. (A) *Nicotiana benthamiana* leaves were transformed transiently with vectors containing cDNAs of *Mla10-cLUC*, *Mla10Lrr22-cLUC* or *Mla22Lrr10-cLUC* together with vectors containing cDNAs of *AVR*_*a10*_-*nLUC* or *AVR*_*a10*_*-V/ AVR*_*a22*_*-V nLUC* lacking signal peptides (SPs), under the control of a 35S promoter. LUC activity was determined forty hours after transfection. The experiment was performed on at least three independent days with two to four replicates (independent set of plants) each day. Significant differences between *AVR*_*a10*_-*nLUC* or *AVR*_*a10*_*-V/ AVR*_*a22*_*-V nLUC* were analyzed using one-way Kruskal-Wallis (KW) analysis. Calculated KW *p*-values are as follows: Mla10: *p* = 0.0001491; Mla22Lrr10: *p* = 0.009035; Mla10Lrr22: *p* = 0.8079. Samples labeled with different letters differed significantly (p < 0.05). (B) Protein levels of MLA10-cLUC, MLA22LRR10-cLUC and MLA10LRR22-cLUC in *N*. *benthamiana* leaf extracts. Proteins were separated on a 8% SDS-PAGE gel and a detected using anti-LUC western blot (WB). (C) Yeast was co-transformed with cDNAs of N-terminal LexABD-fused MLA and N-terminal B42AD-fused AVR_A_ variants. Growth on media lacking Leucine indicates association of respective proteins fused to AD (activation domain) and BD (Binding domain). (D) Protein levels of LexABD-MLA and B42AD-AVR_A_ variants. Proteins were precipitated using an ammonium-acetate buffer and dissolved in a urea-SDS sample buffer before separation on a 10% or 12% polyacrylamide gel and detection by either α-LexA or α-HA WB.

An association between MLA10 and AVR_A10_ was previously shown to be detectable in plant extracts and in yeast [11]. Using a previously established split-LUC complementation assay, we therefore tested, whether the MLA22LRR10 hybrid receptor also specifically interacts with the AVR_A10_ effector when co-expressed *in planta*. We generated constructs expressing *AVR*_*a*_ variants fused C-terminally to the N-terminal part of the LUC reporter (*AVR*_*a*_*-nLUC)*; and *Mla* variants fused C-terminally to the C-terminal part of the LUC reporter (*Mla-cLUC*) [11]. We then performed *A*. *tumefaciens*-mediated transformation of *N*. *benthamiana* leaves to express *AVR*_*a10*_*-nLUC* or *AVR*_*a10*_*-V/AVR*_*a22*_*-V-nLUC* (not recognized *AVR*_*a10*_ variant) together with either *Mla10-cLUC*, *Mla22Lrr10-cLUC* or *Mla10Lrr22-cLUC*. Forty hours post infiltration, we determined LUC activity as a proxy for AVR_A_/MLA association, as described previously [11]. LUC activity was significantly higher in samples that co-expressed *AVR*_*a10*_*-nLUC* with *Mla10-cLUC* or *Mla22Lrr10-cLUC*, when compared to samples where *AVR*_*a10*_*-nLUC* was exchanged with its virulent variant *AVR*_*a10*_*-V/AVR*_*a22*_*-V*. This was not the case when the *AVR*_*a10*_*-nLUC* variants were co-expressed with *Mla10Lrr22-cLUC* (Fig 7A and 7B). Our data suggests a reduced association of AVR_A10_ with the MLA22LRR10 hybrid receptor compared to wild-type MLA10, which is in agreement with differences in cell death scores of *N*. *benthamiana* leaves co-expressing *AVR*_*a10*_*-mYFP* with *Mla10-4xmyc* and *Mla22Lrr10-4xmyc* (S14D Fig).

We also tested whether AVR_A10_ can interact with MLA22LRR10 in the absence of other plant proteins in yeast. We co-expressed *LexABD-Mla10Lrr22* or *LexABD-Mla22Lrr10* under the control of a constitutive ADH1 promoter with *B42AD-AVR*_*a10*_, *B42AD-AVR*_*a10*_*-V/AVR*_*a22*_*-V*, or *B42AD-AVR*_*a22*_ under the control of a galactose (GAL1)-inducible promoter. Co-expressing *B42AD-AVR*_*a10*_ with *LexABD-Mla22Lrr10* or with *LexABD-Mla10* in a yeast two-hybrid assay (Y2H) led to yeast growth on leucine-deprived media (Fig 7C). Little growth was detectable when *LexABD-Mla22Lrr10* or *LexABD-Mla10* was co-expressed with *B42AD-AVR*_*a10*_*-V/AVR*_*a22*_*-V*, while no growth was detected when co-expressing *B42AD-AVR*_*a22*_ (Fig 7C), even though all effector and receptor fusion proteins were detectable in yeast extracts (Fig 7D). Taken together, our findings suggest that the MLA10 LRR domain is responsible for specific recognition of AVR_A10_ and that this is dependent on effector-receptor association. However, we were unable to detect an interaction of *LexABD-Mla22* or *LexABD-Mla10Lrr22* with *B42AD-AVR*_*a22*_ in this Y2H assays (Fig 7C).

### Multiple residues in AVR_A10_ and AVR_A22_ are responsible for differential recognition specificities of MLA10 and MLA22

**Fig 8:**
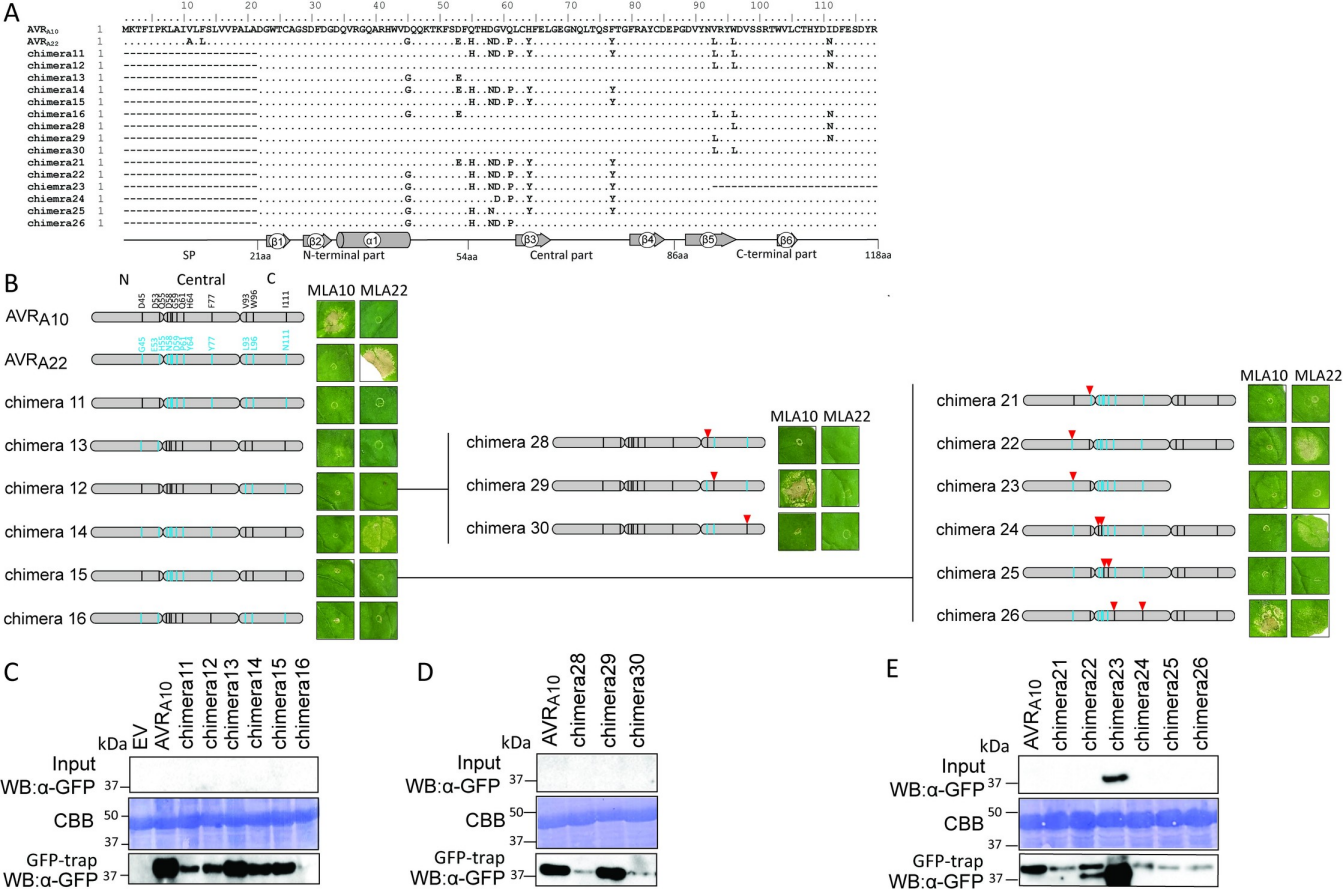
**Co-expression of AVR**_**A10**_ **and AVR**_**A22**_ **chimeras with MLA10 and MLA22 in *N*. *benthamiana*.** (A) Protein sequence alignment of the AVR_A10_/AVR_A22_ chimeric construct. Grey boxes below sequences show the length of the N-terminal, the central and the C-terminal effector part. Dashes represent missing/deletion of effector parts and points designate identical amino acid residues (B) Co-expression of *N*. *benthamiana* with cDNAs of *AVR*_*a10*,_ *AVR*_*a22*_, or chimeric *AVR*_*a10*_*/AVR*_*a22*_ constructs fused C-terminally with mYFP and *Mla10* or *Mla22* cDNAs fused C-terminally with a 4xmyc tag from the 35S promotor in *N*. *benthamiana* leaves. Cell death was scored five days post infiltration and Figures show a representative of at least seven co-transformations. (C-E) Protein levels of AVR_A_-mYFP and chimeric AVR_A10_/AVR_A22_mYFP variants after total protein extraction from *N*. *benthamiana* leaves at two dpi. Proteins were separated on a 12% polyacrylamide gel and detected by α-mYFP western blotting.

It has been proposed that direct fungal effector-plant NLR receptor interactions are mediated by cumulative binding of multiple effector aa residues to the surface of its corresponding NLR receptor [27,39]. We aimed to resolve which of the 11 amino acid residues that are polymorphic between AVR_A10_ and AVR_A22_ alleles (excluding the SP) are responsible for the specific recognition by the cognate MLA10 and MLA22 receptors. On the basis of AVR_A10_ secondary structural predictions, we divided AVR_A10_/AVR_A22_ proteins into three equally long parts: an N-terminal (residues 22–54 aa, comprising ß1-ß2 sheets and the α1-helix, which included the two amino acid substitutions D45G, D53E), a central (55–86aa; comprising the ß3-ß4 sheet and cluster of most amino acid differences Q55H, D58N, G59D, Q61P, H64Y, and the residue F77Y), and a C-terminal part (87–118 aa; including the ß5-ß6 sheets and three amino acid differences V93L, W96L, I111N) (Fig 8A). These individual regions were exchanged between AVR_A10_/AVR_A22_ effector peptides and we then tested the interactions of the six resulting chimeric AVR_A10_/AVR_A22_ effector constructs (called chimera11, chimera12, chimera13, chimera14, chimera15 and chimera16) with MLA10 and MLA22, in *N*. *benthamiana* as described above. All chimeric proteins were detectable after GFP-Trap enrichment except for chimera16-mYFP, which was not consistently detectable (Fig 8C). Co-expression of *chimera14-mYFP* with *Mla22-4xmyc* led to cell death (Figs 8B, S13 and S14). These findings suggest that the C-terminal polymorphic residues V93, W96, and I111 are not responsible for the specific recognition by MLA22.

While in chimera14-mYFP, the three C-terminal residues of AVR_A22_ are exchanged for the respective amino acids found at these positions in AVR_A10_, in chimera12-mYFP the three C-terminal residues of AVR_A10_ are exchanged for AVR_A22_*-*specific amino acid residues (Fig 8A). Even though both effector chimeras were stable *in planta*, chimera12-mYFP was not recognized by MLA10-4xmyc (Figs 8B, 8C and S13 and S14). This suggests that single, double, or triple amino acid mutations at aa positions 93, 96, or 111 within the C-terminus of AVR_A10_ can lead to a loss of MLA10-specific recognition. We sought to determine which amino acid residues at the C-terminus, if exchanged to the respective AVR_A22_ residues, would result in abrogation of MLA10-specific recognition. To this end, we introduced L93V, L96W, or N111I single amino acid substitutions in chimera12-mYFP. The resulting *chimera28-mYFP* (L93V), *chimera29-mYFP* (L96W), and *chimera30-mYFP* (N111I) constructs were co-expressed with *Mla10-4xmyc* and with *Mla22-4xmyc*. Co-expression of *chimera29-mYFP* with *Mla10-4xmyc* led to cell death, while co-expression of *chimera28-mYFP* or *chimera30-mYFP* with *Mla10-4xmyc* did not (Figs 8B–8D and S13 and S14), suggesting that the tryptophan at position 96 in AVR_A10_ is important for MLA10-specific recognition. However, we cannot exclude that differences in protein stability between mYFP fused chimera 29 and 28, or 30, accounts for the lack of cell death in samples co-expressing *chimera28-mYFP* and *chimera30-mYFP* together with *Mla10-4xmyc*.

Even though chimera14-mYFP and chimera15-mYFP differ by only two N-terminal amino acids at positions 45 and 53 and both proteins are detectable, co-expression of *chimera15-mYFP* with *Mla22-4xmyc* did not trigger cell death (Figs 8B, 8C and S13 and S14). This suggests that residues at one or both of these N-terminal positions are necessary for the specific recognition by MLA22. To test this hypothesis, we introduced single D45G or D53E substitutions into *chimera15-mYFP* (Fig 8A). The resulting *chimera21-mYFP* (D53E) and *chimera22-mYFP* (D45G) constructs were co-expressed with *Mla10-4xmyc* or *Mla22-4xmyc*. Co-expression of *chimera22-mYFP* but not *chimera21-mYFP* with *Mla22-4xmyc* led to cell death, while no cell death was observed upon co-expression of *chimera21-mYFP* with *Mla22-4xmyc*, suggesting that the glycine at position 45 but not the glutamic acid at position 53 is essential for MLA22-specific recognition (Fig 8A, 8B and 8E).

To test if the N-terminal and central parts of the *AVR*_*a22*_ effector are sufficient to trigger cell death when co-expressed with *Mla22*, we constructed a deletion construct (Δ93–118) of *chimera22*, which we termed *chimera23* (Fig 8A). Co-expression of *chimera23-mYFP* with *Mla10-4xmyc* or *Mla22-4xmyc* did not trigger cell death, although the chimera23-mYFP protein was seemingly more stable than AVR_A10_ and all other chimeric proteins (Figs 8B–8E and S13 and S14). Even though the C-terminal residues L93, L96, and N111 are not specifically recognized by MLA22, our findings suggest that the C-terminal region of the AVR_A22_ effector potentially stabilizes the conformation of the N-terminal and central regions, which are necessary for MLA22-specified recognition. In summary, the N-terminal glycine at position 45 and the C-terminal tryptophan at position 96 are important for MLA22- and MLA10-specific recognition, respectively.

In addition, we assessed the role of amino acids in the central positions 55, 58, 59, 61, 64, and 77 for MLA10- and MLA22-specific recognition. We introduced double mutations in *chimera22-mYFP* to generate *chimera24-mYFP* (H55Q and N58D), *chimera25-mYFP* (D59G and P61Q) and *chimera26-mYFP* (Y64H and Y77F), which were co-expressed with *Mla10-4xmyc* or *Mla22-4xmyc* (Fig 8A). While co-expression of *chimera24-mYFP* with *Mla22-4xmyc*, but not with *Mla10-4xmyc* led to a specific cell death response, co-expression of *chimera25-mYFP* with *Mla22-4xmyc* or with *Mla10-4xmyc* did not lead to cell death in *N*. *benthamiana* leaves (Figs 8B–8E and S13 and S14). Surprisingly, *chimera26-mYFP* triggered a strong cell death response when co-expressed with *Mla10-4xmyc* and a subtle but consistent cell death phenotype when co-expressed with *Mla22-4xmyc*, suggesting that it is recognized by both receptors (Fig 8B–8E).

**Fig 9:**
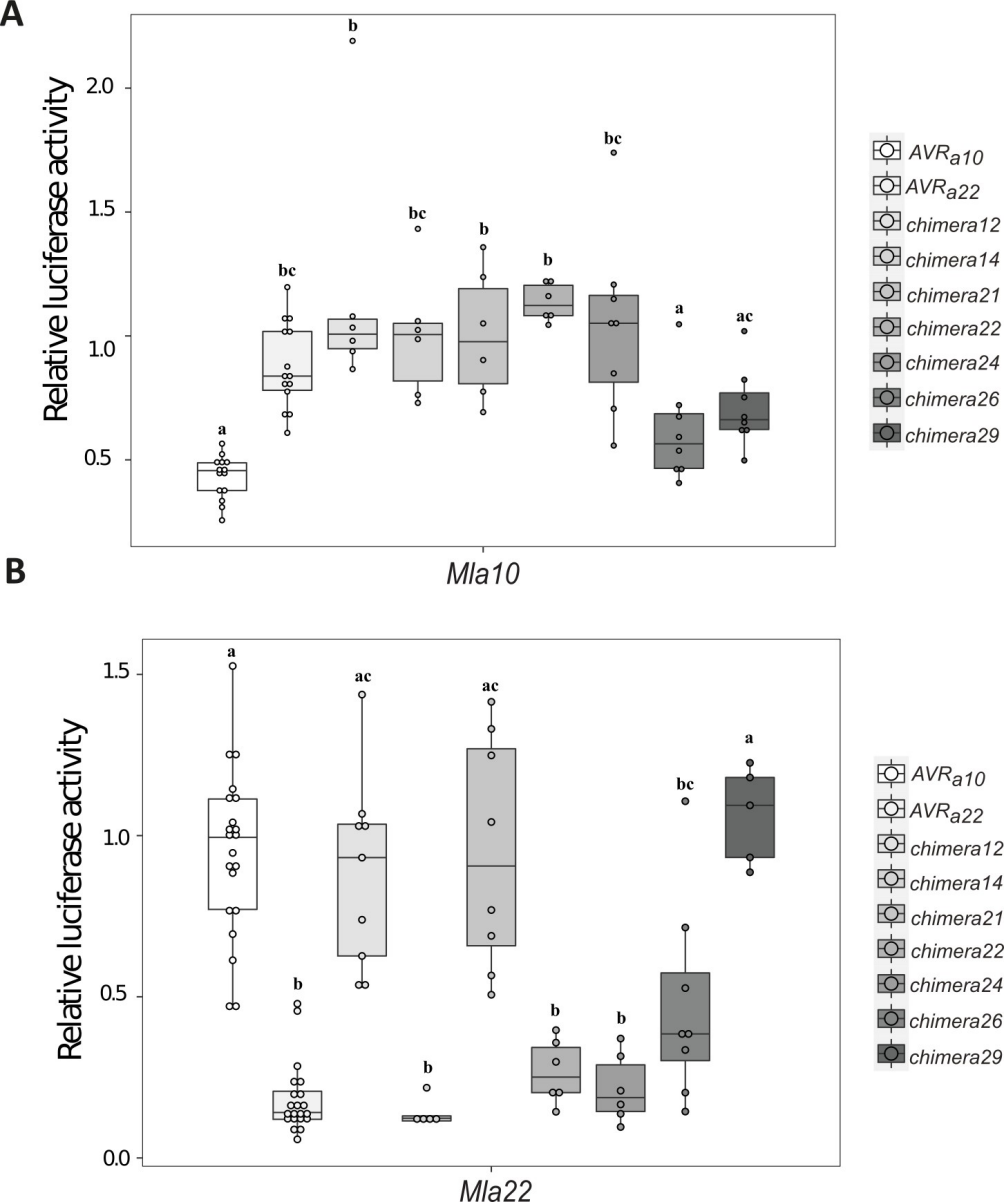
Co-expression of a selection of AVR_A10_/AVR_A22_ chimeras with MLA10 and MLA22 in barley protoplasts. Barley protoplasts of *cv*. Golden Promise were co-transfected with a LUC reporter assay and pIPKb002 vectors containing cDNAs of *AVR*_*a10*_, *AVR*_*a22*_, *chimera12*, *chimera14*, *chimera21*, *chimera22*, *chimera24*, *chimera26* or *chimera29* without signal peptide or with an empty vector (EV) together with either (A) *Mla10* or (B) *Mla22* in pipkb002. Transfections were performed at least six times independently. Significant differences between samples were analyzed using non-parametric Kruskal-Wallis (KW) analysis followed by the Dunn’s test. Calculated KW *p*-value are as follows: *Mla10*: *p* = 1.439e-07, *Mla22*: *p* = 5.374e-11. Samples labeled with different letters differed significantly (*p* < 0.05) in the Dunn’s test.

To independently verify the data, we also co-expressed a selection of *AVR*_*a10*_*/AVR*_*a22*_ chimeras (*chimera12*, *chimera14*, *chimera21*, *chimera22*, *chimera24*, *chimera26 and chimera29*) together with *Mla10* or *Mla22* in protoplasts of barley *cv*. Golden Promise and determined cell viability by LUC activity, as described above. LUC activity was approximately 50% lower in samples co-expressing *Mla10* with *AVR*_*a10*_ when compared to samples that co-express *Mla10* with *AVR*_*a22*,_ and this is in agreement with previously published data [11] (Fig 9A). We detected intermediate LUC activity when co-expressing *Mla10* together with *chimera26* or *chimera29*, but this reduced LUC activity did not differ significantly from the sample co-expressing *Mla10* and *AVR*_*a10*_ (Fig 9A). This was not the case for samples co-expressing *Mla10* together with *chimera12*, *chimera14*, *chimera21*, *chimera22*, or *chimera24* (Fig 9A).

LUC activity was on average 80% lower in samples co-expressing *Mla22* with *AVR*_*a22*_ when compared to samples that co-express *Mla22* with *AVR*_*a10*_, and this is again in agreement with published data [11]. LUC activity of samples co-expressing *Mla22* together with *chimera14*, *chimera22*, *chimera24* and *chimera26* was not statistically different from the activity observed when co-expressing *Mla22* and *AVR*_*a22*_ (Fig 9B). This was not the case for samples co-expressing *Mla22* together with *chimera12*, *chimera21* or *chimera29*. Notably, we detected an intermediate relative LUC activity when co-expressing *Mla22* together with *chimera26* (Fig 9B). Similarly, cell death scores of *N*. *benthamiana* leaves co-expressing *Mla22-4xmyc* together with *chimera26-mYFP* were also lower than when co-expressing *Mla22-4xmyc* together with *AVR*_*a22*_*-mYFP* (Figs 8 and S14). We thus conclude that the barley protoplast cell death data (Fig 9) overall recapitulate the MLA10 and MLA22 specificities towards AVR_A_ chimeric constructs observed in the heterologous *N*. *benthamiana* system (Fig 8).

In summary, our findings suggest that for triggering MLA10-specific cell death, the four residues D53, H64, F77, and W96 of AVR_A10_ cannot be exchanged to the residues found in AVR_A22_. In turn, to trigger MLA22-specific cell death, the five amino acid residues G45, H55, N58, D59, and P61 of AVR_A22_ cannot be exchanged to the residues found in AVR_A22._ Furthermore, deletion of the C-terminal third of the AVR_A10_ and AVR_A22_ effectors leads to loss of avirulence function (Fig 8).

**Fig 10:**
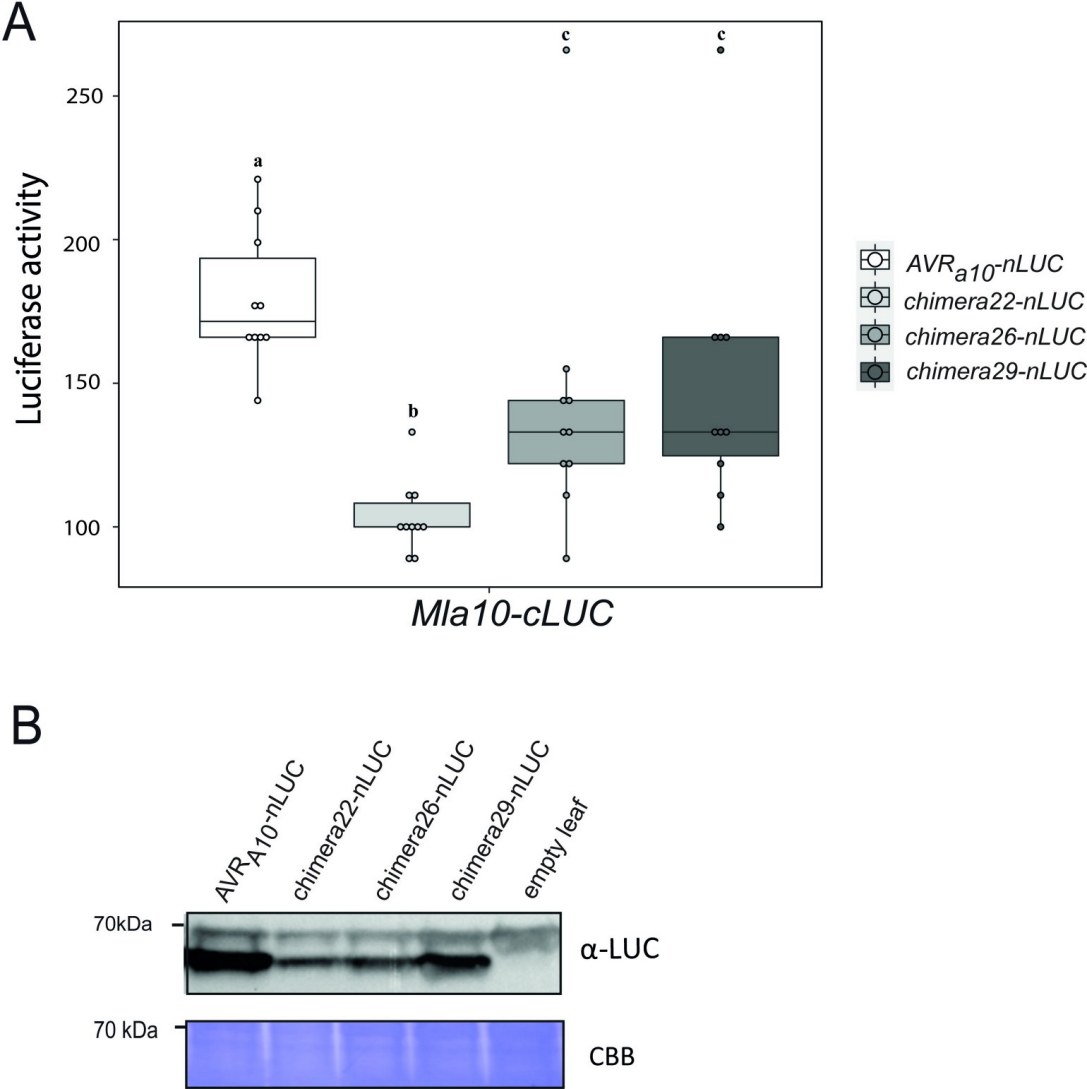
AVR_A10_ amino acid residues that are responsible for specific recognition correlate with residues that interact with the MLA10 receptor. (A) *N*. *benthamiana* plants were transformed transiently with vectors containing cDNAs of *Mla10-cLUC* together with cDNAs of *AVR*_*a10*_*-nLUC* without signal peptide, *chimera22-nLUC*, *chimera26-nLUC* or *chimera29-nLUC* under the control of a 35S promoter. LUC activity was determined 40 h after *A*. *tumefaciens*-mediated transformation. The experiment was performed on at least four independent days with two to four replicates (independent set of plants) each day. Significant differences between samples were analyzed using non-parametric Kruskal-Wallis (KW) analysis followed by the Dunn’s test. Calculated KW *p*-value = 5.03e-05. Samples labeled with different letters differed significantly (*p* < 0.05) in the Dunn’s test. (B) Protein levels of AVR_A10_-nLUC, chimera22-nLUC, chimera26-nLUC and chimera29-nLUC. Proteins were separated on a 8% SDS_PAGE gel and a detected using anti-LUC western blotting (WB).

To determine if MLA-mediated cell death initiated by recognition of the AVR_A_ effector also correlates with receptor-effector association in plant extracts, we again applied the split-LUC complementation assay. We transiently expressed *Mla10-cLUC* together with *AVR*_*a10*_*-nLUC*, *chimera26-nLUC*, *chimera29-nLUC* and as a control, *chimera22-nLUC* in *N*. *benthamiana* leaves, followed by LUC measurements at 40 hours post infiltration of leaves with the *A*. *tumefaciens* carrying constructs of interest. LUC activity of samples co-expressing *Mla10-cLUC* with *AVR*_*a10*_*-nLUC*, *chimera26-nLUC* and *chimera29-nLUC* was significantly higher than the LUC activity observed in the samples expressing *Mla10-cLUC* together with *chimera22-nLUC* (Fig 10A and 10B). Chimera26 and chimera29 but not chimera22, can trigger MLA10-mediated cell death in co-expression assays (Figs 8 and 9), and as such, we conclude that the recognition specificities mediated by MLA10 towards the AVR_A_ chimeric variants 26, 29 and 22 correlate with receptor-effector association. We again only observed intermediate levels of LUC activity in samples co-expressing *Mla10-cLUC* together with *chimera26-nLUC* or *chimera29-nLUC* (Fig 10A). This is in agreement with the quantitative cell death assay in barley protoplasts (Fig 9A), and suggests that when compared to *AVR*_*a10*,_ these constructs are impaired in their ability to activate and associate with MLA10 *in planta* and this may be associated with levels of protein expression (Fig 10B).

### Two amino acids of AVR_A10_ that cannot be exchanged to respective AVR_A22_ residues are located in a predicted positively charged area that corresponds to the catalytic cleft of the fungal F1 RNase

**Fig 11:**
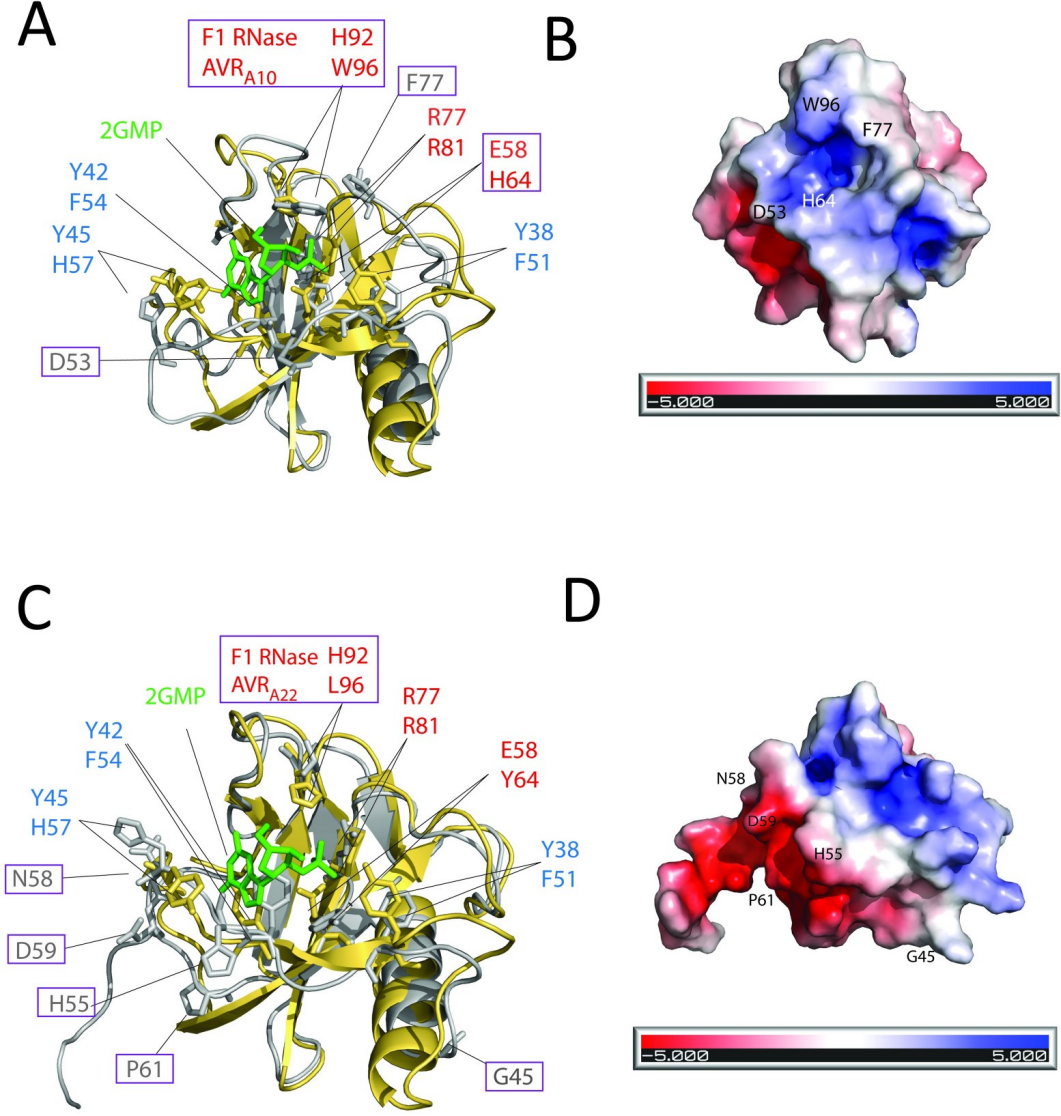
The location of amino acid residues in AVR_A10_ and AVR_A22_ that determine MLA10 and MLA22 recognition specificities. (A and C) show structural superimposition of the crystal structure of *Fusarium moniliformis* RNase F1 (yellow) and IntFOLD version 5.0 structural predictions of AVR_A10_ or AVR_A22_ (grey). Depicted in green is the F1 RNase ligand 2’-guanosine monophosphate (2’ GMP); residues of the F1 RNase catalytic triad and corresponding AVR_A_ residues are depicted in red; residues of the F1 RNase RNA binding pocket and corresponding AVR_A_ residues are shown in blue. The residues of AVR_A10_ and AVR_A22_ required for specific MLA10 and MLA22 recognition as determined in Figs 8–10 are framed with a purple rectangle. (B and D) Predicted electrostatic surface potential of the AVR_A10_ and AVR_A22_ effector surface calculated using Adaptive Poisson Boltzmann Solver (APBS) [67]. The residues of AVR_A10_ and AVR_A22_ required for specific MLA10 and MLA22 recognition as determined in Figs 8–10 are indicated. Below is a scale bar of the electrostatic potential (red = negative charge, white = neutral charge, blue = positive charge).

Microscale thermophoresis assays suggested that CSEP0064 has some affinity to total RNA but its X-ray structure suggests that it lacks residues required for RNA hydrolysis [30]. Our data also suggests that the putative RNase-like fold of AVR_A_ effectors is not associated with RNase activity (Fig 4). Here, we examined the location of the AVR_A10_ and AVR_A22_ residues that are required for specific recognition by MLA10 and MLA22, and if the corresponding residues in the F1 RNase are required for RNA binding and hydrolysis. To do this, we superimposed AVR_A10_ and AVR_A22_ predicted structures on the structure of *Fusarium moniliformis* F1 RNase. Residues Y38, Y42, and Y45 of the F1 RNase are involved in binding the ribose and phosphate of 2’ GMP and the respective amino acids found in AVR_A10_ (F51, F54, and H57) are identical to those of AVR_A22,_ and as such, do not account for specific MLA recognition (Fig 11A–11C). The R77 residue in the F1 RNase, also forms a contact with the phosphate in 2’ GMP. The corresponding residue can also be found in AVR_A10_ and AVR_A22_ (residue R81, Fig 11A–11C) but not in the CSEP0064 structure or the predicted structures of any other AVR_A_ effector isolated so far. The residues W96 and H64 of AVR_A10_ are L96 and Y64 in AVR_A22_ and have dissimilar properties to the corresponding residues (H92 and E58, respectively) in the F1 RNase. Electrostatic potential prediction using Adaptive Poisson-Boltzmann Solver (APBS) of surfaces suggests that H64, R77, and W96 in AVR_A10_ and L64, R77, and L96 in AVR_A22_ belong to a positively charged cleft (Fig 11B–11D). In AVR_A10_, these residues are predicted to be part of the positively charged surface patch. For recognition by MLA10, H64 and W96 can indeed not be exchanged to the respective residues found in AVR_A22_ (Fig 11B). In contrast, the AVR_A22_ residues that cannot be exchanged to the respective AVR_A10_ residues without losing MLA22 avirulence activity, can be found in a negatively charged surface patch away from the positively charged area, presumably required for MLA10 recognition (Fig 11D). These results suggest that the AVR_A_ residues, which confer specific recognition by MLA10 and MLA22 receptors, are located in distinct predicted surface patches of the avirulence proteins encoded by allelic *AVR*_*a10*_ and *AVR*_*a22*_.

## Discussion

### Identification of AVR_A6_

Long-read DNA sequencing and high-quality genome assembly of the DH14 *Bgh* isolate recently recovered 30 Mb of previously unassembled repetitive regions of the *Blumeria* genome, which linked together multiple non-contiguous series of genomic sequences, including scaffold 16 [40]. This may explain why we failed to identify BLGH_00708, BLGH_00709 and BLGH_07091 as AVR_A6_ in our previous studies [11,28] and emphasizes the importance of high-quality *Blumeria* genome assemblies for identification of novel AVR_A_ effectors. We found three *AVR*_*a6*_ paralogues in the RACE1 genome, and in line with this observation, three non-identical paralogues of *AVR*_*a7*_ have been described in RACE1 [11], indicating that these *AVR*_*a*_ effectors were duplicated [11]. Similarly, *BLGH_07092* is likely an *AVR*_*a6*_ copy with a frameshift mutation. This *AVR*_*a*_ duplication could facilitate the gain of new virulence functions.

Unlike other virulent variants of *Bgh AVR*_*a*_ effectors that carry SNPs in the coding region or transposon insertions, or are transcriptionally silent [11,28], here we report that virulence of *Bgh* isolates on *Mla6* NILs is possibly caused by splice site mutations in the intron of transcripts from isolates virulent on *Mla6* lines that map to the *AVR*_*a6*_ gene. The intron retention observed in most of these transcripts is potentially caused by either a splice branch point mutation or a splice donor site mutation and leads to premature stop codons (S3 and S4 Figs).

Even though the encoded truncated proteins are detectable in heterologous *N*. *benthamiana* (Fig 2C), RNA-Seq analysis of virulent *Bgh* isolates on barley leaves suggests that these mutations result in significantly reduced transcript levels. The latter is possibly a consequence of nonsense-mediated mRNA decay (NMD) [41]. This observation, together with a high virulence frequency on Mla6 NILs in European *Bgh* populations [42], suggests that the loss of the *AVR*_*a6*_ effector gene does not have a detrimental impact on *Bgh* virulence. Future studies might clarify whether *AVR*_*a6*_ virulence function can be compensated by *AVR*_*a6*_ family members or more distantly related effectors.

### A predicted common ribonuclease-like fold among *Blumeria* AVR_A,_ AVRPM3 and AVRPM2 effectors

AVR_A6_ was predicted to be structurally similar to CSEP0064 (Fig 3). Upon re-analysis of all previously reported *Bgh* AVR_A_ effectors using the version 5.0 of the IntFOLD structural prediction server, we report here evidence supporting a common structural fold amongst the so far isolated AVR_A_ effectors despite the lack of relatedness between their DNA and protein sequences (Fig 3). Moreover, AVR_A_ effectors appear to share this structural fold at least with *Bgt* AVRPM2 and AVRPM3^D3^ effectors that are recognized by wheat NLRs PM2 and PM3, respectively (Fig 3; [22,35,36]). In addition, protein alignments and *in silico* tertiary structure modelling suggest that *Bgt* AVRPM3^A2/F2^ and AVRPM3^B2/C2^ also have a central α-helix that faces three to four β-sheets [22]. Even though AVR_A_ effectors seem to have a common predicted fold similar to RNases, the residues critical for catalytic activity are lacking in the predicted AVR_A_ structures and we did not detect ribonuclease activity when AVR_A_ effectors were co-incubated with RNA (Fig 4A).

Given that multi-allelic barley MLA, multi-allelic wheat PM3, and wheat PM2 NLRs are sequence-unrelated and are encoded on non-syntenic chromosomal locations, it is possible that these immune receptors arose by convergent evolution to detect distinct members of the structurally related superfamily of powdery mildew RALPH effectors.

We speculate on two evolutionary scenarios that might explain the diversity of extant RNase-like effectors in *Bgh* and *Bgt*. In a common descent scenario, all RALPH effectors have diversified from an “*ur*-RALPH”, which was present in the last common ancestor prior to host specialization of grass powdery mildews [29]. For instance, striking sequence conservation of *AvrPm2* among *Blumeria ff spp secalis*, *tritici* and *hordei* [36] and the presence of orthologous candidate effector gene families among specialized forms of grass powdery mildews support a common descent model [43]. The relatively small number of RALPH effectors in dicot-infecting powdery mildew species such as *Erysiphe pisi* [44] and in the early-diverged *Parauncinula polyspora* [45] suggests that RALPHs could have evolved 80–90 million years ago [46]. However, the variable intron location in *Blumeria AVR* genes (S6 and S7 Figs) questions this hypothesis. In an alternative scenario, the predicted structural relatedness of known AVRPM and AVR_A_ effectors could be the product of convergent evolution from different fungal RNases in the order Erysiphales after the differentiation of *formae speciales*. In summary, our data is reminiscent of findings indicating the existence of sequence unrelated but structurally related MAX effectors (*Magnaporthe* Avrs and ToxB-like) that account for 10% of the *M*. *oryzae* effector repertoire in this Ascomycete pathogen [47]. Another example for sequence-diversified but structurally-related effectors are oomycete RXLR effectors, such as ATR1 and ATR13 in *Hyaloperonospora arabidopsidis* [48,49], and PexRD2 and AVR3a11 in *Phytophthora infestans* [50].

### The LRRs of allelic MLA receptors determine recognition of matching AVR_A_ effectors

Earlier work with hybrids built from MLA1 and MLA6 receptors demonstrated that the MLA LRR is a determinant of *Bgh* isolate-specific recognition [38]. In this previous study it could not be clarified whether the respective AVR_A_ effectors are the only fungal components that determine isolate-specific recognition by the MLA LRR domains. We show here that the LRR of four MLA receptors (MLA1, MLA6, MLA10, and MLA22) is responsible for specific recognition of matching AVR_A_ effectors. We found that for the recognition of AVR_A6,_ the C-terminal six LRR repeats of MLA6 cannot be exchanged to the ones found in MLA1, while the 12 C-terminal LRR repeats of MLA1 cannot be exchanged to those of MLA6 for recognition of AVR_A1_. Similarly, four C-terminal LRR repeats of the flax allelic L5 and L6 receptors are necessary for recognition of matching AvrL567 effectors [27]. Most sites of positive selection among MLA resistance specificities cluster on the predicted concave site of the C-terminal LRRs [19,20]. Thus, it is possible that these LRR residues are contact sites for specific recognition and association with structurally related AVR_A_ effectors. We confirmed this assumption for the LRRs of MLA10 by observing an association of MLA22LRR10 with AVR_A10_ when the proteins were co-expressed in plants or in yeast (Fig 6C).

Unexpectedly, whereas M11166, M16666, M61111, and M66111 chimeras clearly recognize the corresponding AVR_A_ effectors in barley protoplasts (Fig 5A), the latter three hybrid receptors are non-functional in heterologous *N*. *benthamiana*. The resistance function of several barley *Mla* recognition specificities, including *Mla6*, is genetically dependent on *HvRar1*, *HvSgt1*, and *HvHsp90* [38,51,52], which form a chaperone/co-chaperone complex in which *Hv*HSP90 directly interacts with the LRR of MLA [52,53]. Thus, the hybrid MLA1/MLA6 receptors might be dependent on an additional barley protein for full resistance function. Interestingly, the function of M11166 is known to be fully independent of *HvSGT1* and *HvRAR1* in barley and we have shown here that this is the sole MLA6/MLA1 chimeric receptor functional in *N*. *benthamiana* [38,51].

### Multiple polymorphic AVR_A10_/AVR_A22_ residues influence recognition by MLA10 and MLA22

*AVR*_*a10*_ and *AVR*_*a22*_ effector alleles are maintained as a balanced polymorphism in a worldwide collection of *Bgh* isolates [11], which implies an important virulence function for the *AVR*_*a10*_/*AVR*_*a22*_ gene, supported by its membership in the *Blumeria* core effectorome [40]. As the effector alleles with only 11 polymorphic amino acids likely adopt an identical protein structure, we aimed here to pinpoint polymorphic residues in AVR_A10_/AVR_A22_ recognized by MLA10 and MLA22, respectively. Some effector chimeras, including chimera11 or chimera13 containing only two polymorphic residues compared to the respective WT avirulence effectors, escaped recognition by MLA10 and MLA22, even though these are stable proteins in *N*. *benthamiana*. This is consistent with the observation that other naturally occurring virulent AVR_A_ effector variants can escape recognition by only one or two amino acid substitutions in the respective AVR_A_ polypeptides [11,28]. Similarly, one amino acid exchange in *Bgt* AVRPM3^A2/F2^ leads to a loss of pathogen recognition [54]. We identified four residues in AVR_A10_ that cannot be exchanged to the ones found in AVR_A22_ without losing recognition by MLA10. Four different residues in AVR_A22_ cannot be exchanged to the ones found in AVR_A10_ without losing recognition by MLA22. Of note, chimera26 can be recognized by MLA10 and MLA22 (Figs 8 and 9), further suggesting that differential regions in AVR_A10_ and AVR_A22_ are recognized by MLA10 and MLA22, respectively. Our findings are consistent with the identification of multiple residues, spread along the *Bgt* AVRPM3^B2/C2^ effector polypeptide, that cannot be exchanged for specific detection by wheat PM3b or PM3c NLRs [22]. Multiple, additive contact points of the AvrL567-A and -D flax rust fungus alleles are recognized by the flax receptors L5 and L6, respectively [27]. In summary, our findings suggest that multiple residues on the AVR_A_ effector surface determine the specific recognition by MLA receptors, and this may influence the functional diversification process of these receptors.

Previous studies suggested that RALPH effectors are pseudoenzymes that cannot cleave RNA due to the absence of a catalytic amino acid triad present in the fungal F1 RNase that are needed for enzymatic RNA catalysis [30]. These catalytic residues are E58, R77, and H92 [55]. We found that only one residue involved in RNA catalysis (R81) is conserved in the deduced AVR_A10_/AVR_A22_ RNase-like effectors. Notably, four amino acids in AVR_A10_ cannot be exchanged to the ones found in AVR_A22_ (D53, F77, H64, and W96), and these are located close to a predicted positively charged area on the effector’s surface. In the F1 RNase, the corresponding area forms the catalytic cleft. Residues required for MLA22 recognition are found in a negatively charged surface patch away from the negatively charged area presumably recognized by MLA10 (Fig 11A and 11B). This data is underlined by the recognition of chimera26 through MLA10 and MLA22, as chimera26 carries both of the described recognition patches. Recently, a few RALPH effectors were found in *E*. *pisi*, which infects a dicotyledonous host, and structural predictions showed that residues for RNA catalysis are partially conserved and are located within a positively charged binding cleft [44]. This is in agreement with our findings for *Bgh* AVR_A10_ and AVR_A22_ but contrasts with the absence of any catalytic triad residue as well as a positively charged binding cleft in the structure of *Bgh* CSEP0064 [30]. If these residues, which are located in the predicted positively charged cleft, and are recognized by MLA10, are also involved in potential RNA binding of AVR_A10_ and AVR_A22_ remains to be determined.

## Methods

### Phylogenetic analysis of *Blumeria graminis* formae speciales candidate-secreted effector proteins

Secretomes for the *B*. *graminis* formae speciales *poae*, *lolium*, *avenae*, *tritici* 96224, *hordei* DH14, *secalis* S1459, *triticale* T1-20, and *dactylidis* were obtained as described in Frantzeskakis et al. 2019 [40]. Subsequently, protein sequences without the signal peptide were aligned using MAFFT v7.310 (command used: mafft—amino —6merpair—maxiterate 1000—thread 12; [56]). The resulting alignment was then passed to IQ-TREE v1.6.beta4 (command used: iqtree-1.6.beta4-Linux/bin/iqtree -m VT+R8 -s all_seqs.fa.aln -nt 12 -bb 1000; [57]), and the phylogenetic tree generated was visualized using ITOL (https://itol.embl.de/tree/13461102183294661576347461; [58]). If not already publicly available [40,43,59], proteomes used for secretome prediction were generated using the MAKER pipeline [60] as described previously [40].

### Plant material

The barley cultivar Golden Promise was grown at 19°C, 70% humidity and under a 16 h photoperiod. *N*. *benthamiana* plants were grown and maintained under standard greenhouse conditions.

### Association analysis

RNA-seq read alignment, variant calling, and association analysis were performed as described in Saur *et al*., 2019 [11].

### Generation of expression vectors

Entry clones and destination constructs for the expression of *AVR*_*a1*_, *AVR*_*a1*_*-V1*, *AVR*_*a10*_, *AVR*_*a22*_, *AVR*_*a10*_*-V/AVR*_*a22*_*-V*, *Mla10*, *Mla22*, *Mla1*, *and Mla6* were previously published by Saur et al., 2019 [11]. *CSEP0058* (*BLGH_00697*) was cloned from the cDNA of *Bgh* isolate DH14 using the primers listed in S3 Table. *M16666*, *M61111*, *M11166*, and *M66111* DNA sequences with introns in the pUBI-NOS vector were previously published by Shen et al., 2003 [38], and for expression in *N*. *benthamiana* were amplified from the pUBI-NOS vector [38] and cloned into pENTR/D-TOPO without a stop codon (S3 Table). cDNAs of *AVR*_*a6*_, *AVR*_*A6*_*-V2*, *CSEP0333* (*BLGH00698*), and *BLGH_00700*, *chimeras 11*, *12*, *13*, *14*, *15*, *16*, and *23*, chimeric *Mla10Lrr22* and *Mla22Lrr10* were synthesized with or without a stop codon as pDONR221 (Km^R^) entry clones by GeneArt (Thermo Fisher). *Chimeras 21*, *22*, *24*, *25*, *26*, *28*, *29*, and *30* were generated by site-directed mutagenesis PCR using primers listed in S3 Table. The integrity of all entry clones was confirmed by Sanger sequencing.

For transient expression assays in barley protoplasts, *N*. *benthamiana*, and yeast, the genes were recombined using LR-Clonase II (Thermo Fisher) into the pIPKb002 (*Spec*^*R*^) [61], pGWB517 (*Spec*^*R*^) [62], pXCGS-GW-mYFP (*Carb*^*R*^) [63], the pLexA-GW (*Carb*^*R*^) [64], or the pB42AD-GW (*Carb*^*R*^) [64] gateway-compatible destination vectors. Additional constructs used in this study were described in Saur *et al*., 2019 [11].

### Transient gene expression assays in barley protoplasts

The isolation and transfection of barley protoplasts was performed as described in Saur *et al*., 2019 [33]. In short, cDNAs of the *AVR*_*a*_s were co-expressed with cDNAs of *Mla10*, *Mla22*, *Mla10Lrr22*, and *Mla22Lrr10* using the pIPKb002 vector with a strong ubiquitin promoter or with intron-containing DNA of chimeras *M16666*, *M11166*, *M61111*, or *M66111* in a pUBI-NOS-vector (described in Shen et al., 2003 [38]) in barley *cv*. Golden Promise protoplasts. Protoplast solution (300 μl of 3.5 x 10^5^ cells/ml) was transfected with 4.5 μg of *LUC* reporter construct, 10 μg of *Mla* plasmid, and 6.5 μg of the respective *AVR*_*a*_ effector or an empty vector (*EV*). The protoplasts were incubated for 16 h at 21°C in a plant growth chamber and then harvested by centrifugation at 1,000 x *g*. Subsequently, the supernatant was removed, and protoplasts were lysed by addition of 180 μl of cell culture lysis reagent (Promega, E1531). The LUC activity of samples was measured in a luminometer (Centro, LB960) using a 96-well plate in which 50 μl of protoplast lysate were mixed with 50 μl of the LUC substrate (Promega, E1501). The relative LUC units (RLU) were calculated by setting the absolute value of the EV sample to 1.

### Transient gene expression assays in *Nicotiana benthamiana*

Expression constructs for AVR_A_ and MLA and respective chimeras were always freshly transformed into *Agrobacterium tumefaciens* strains GV3101::pm90 and GV3101::pm90RK and selected on LB media containing the respective antibiotic resistance. Single colonies were inoculated into liquid LB medium and grown overnight at 28°C with agitation at 220 rpm to a maximal OD_600_ = 1.5. Agrobacteria were centrifuged at 2500 x *g* for 15 min and the pellet was resuspended in infiltration buffer (10 mM MES, pH 5.6, 10 mM MgCl_2_, and 200 μM acetosyringone) to an OD_600_ of 1 to 1.2. The suspensions of Agrobacteria were incubated at 28°C with shaking 150 rpm for at least 2 h. Leaves of four-week-old *N*. *benthamiana* plants were infiltrated with a 1:1 mix of bacteria carrying *AVR*_*a*_ constructs or *Mla* constructs. The cell death score was assessed at four days post infiltration. Leaf tissue was harvested two days post infiltration for western blot analysis and 40 hours for split-LUC assays.

### Split-luciferase complementation assay

The assay was performed as described in Saur *et al*., 2019 [11].

### Plant protein extraction and immunoprecipitation for detection of fusion proteins

*N*. *benthamiana* leaf material was frozen in liquid nitrogen and ground to a fine powder using a Retsch bead beater.

For the detection of AVR_A_-nLUC and MLA-cLUC proteins, 50 mg of leaf tissue was resuspended in 150 μl of urea-SDS sample buffer (50 mM Tris-HCl pH 6.8, 2% SDS, 8 M urea, 2% β-mercaptoethanol, 5% glycerol, and 0.004% bromophenol blue) and vortexed at room temperature for 10 min before centrifugation at 16,000 × *g f*or 10 min.

For the detection of AVR_A_-mYFP and MLA-4xmyc proteins, 300 mg of ground leaf tissue were dissolved in 2 mL of ice cold extraction buffer (150 mM Tris-HCl, pH 7.5, 150 mM NaCl, 10 mM EDTA, 10% (v/v) glycerol, 10 mM DTT, 2% (v/v) plant protease inhibitor cocktail (Sigma), 1 mM NaF, 1 mM Na_3_VO_4_, 1 mM PMSF, and 0.5% (v/v) IGEPAL). Extracts were centrifuged twice for 16 min at 16,000 x *g* at 4°C. For the detection of MLA-4xmyc proteins, the extracts were diluted 4:1 with 4 x SDS loading buffer for SDS-PAGE. Samples were heated for 5 min at 95°C. For the detection of AVR_A_-mYFP, the proteins were concentrated using GFP-trap-MA (Chromotek) beads. Beforehand, the beads were incubated in equilibration buffer (150 mM Tris-HCl, pH 7.5, 150 mM NaCl, 10 mM EDTA, pH 7.5, 10% Glycerol, 1.5% (w/v) BSA) for 1 hour at 4°C with slow rotation. The protein extracts were incubated with the equilibrated beads for 4 h at 4°C with slow rotation. Subsequently, the beads were washed five times with cold wash buffer at 4°C. The conjugated proteins were stripped off the beads by boiling the samples in 30 μl 4 x Laemmli sample buffer at 95°C for 10 min.

Samples were separated on 8% or 10% SDS-PAGE gels, blotted onto PVDF membranes and detected using anti-LUC (SIGMA L0159), anti-GFP (abcam ab6556) or anti-myc (abcam ab9106) antibodies followed by anti-rabbit IgG-HRP (Santa Cruz Biotechnology sc-2313). Proteins were detected with the SuperSignal West Femto chemiluminescent substrate (Thermo Fisher, catalog number 34095) using Gel Doc XR and a gel documentation system (Bio-Rad).

### Protein expression and purification from *Escherichia coli*

AVR_A6_ (25–115) AVR_A10_ (21–119), and AVR_A13_ (21–122) were expressed in *E*. *coli* as fusion proteins with N-terminal GST tags. The expression plasmids pGEX6p-1 (GE Healthcare) were transformed into the *E*. *coli* strain BL21 (DE3) (Novagen) by heat shock and grown at 37°C in Luria-Bertani broth to an OD_600_ of 0.6. Isopropyl-β-D-thiogalactoside (IPTG, Sigma) was added to induce protein expression at 18°C for a further 12 h. The cells were harvested by centrifugation at 6,000 x *g* for 10 min at 4°C and resuspended in resuspension buffer (25 mM TRIS pH 8, 150 mM NaCl). Cell suspensions were lysed by sonification. Cell debris was removed by centrifugation at 30,000*g* for 2 h. The soluble fractions were collected and allowed to flow through GST resin (GE Healthcare). After washing with two column volumes of the same buffer used for resuspension, another 2 ml of buffer and 10 μl of PreScission protease (GE Healthcare) were added to the column followed by overnight incubation to cleave off the AVR_A_ proteins from the GST resin. The cleaved AVR_A_ proteins were then eluted and further purified by size-exclusion chromatography using a Superdex 200 10/30 gel filtration column (GE Healthcare).

### Protein expression and purification from insect cells

AVR_A6_ (25–115) AVR_A10_ (21–119), and AVR_A13_ (21–122) were expressed in insect cells as fusion proteins with N-terminal GST tags. The expression plasmids pFASTBAC1 (Invitrogen) were transformed into the *E*. *coli* strain DH10Bac (Invitrogen) by heat shock. Successful transformation was validated by blue-white selection and bacmids of positive colonies were subsequently isolated with a DNA Mini Kit (QIAGEN). Sf21 insect cells (Invitrogen) were transfected with sequence verified bacmids by CellfectinII (Thermo Fisher). After five days incubation at 28°C, recombinant baculovirus P0 were harvested and used to amplify P1 virus for another three days. Insect cells were infected at concentration of 2.0 x 10^6^–2.5 x 10^6^ cells/ml with P1 virus for 60 h. Insect cells were harvested and re-suspended in resuspension buffer (25 mM TRIS pH 8, 150 mM NaCl,15mM imidazole) followed by sonification lysis. Cell debris were removed by centrifugation at 30,000*g* for 2 h. The soluble fractions were collected and allowed to flow through a GST affinity trap. After washing with two column volumes of the same buffer used for resuspension, another 2 ml of buffer and 10 μl of PreScission protease (GE Healthcare) were added to the column, followed by overnight incubation to cleave off the AVR_A_ proteins from the GST resin. The cleaved AVRA proteins were then eluted and further purified by size-exclusion chromatography using a Superdex 200 10/30 gel filtration column (GE Healthcare). The proteins were tested for RNase activity with the same method applied for *E*. *coli* purified proteins.

### RNase activity assays

Leaf material from three-week-old barley *cv*. Golden Promise plants was harvested to extract total RNA using the RNeasy Plant Mini Kit (QIAGEN). The remaining genomic DNA was removed by treating RNA with TURBO DNase enzyme (Ambion). Purified AVR_A_ effectors from *E*. *coli* were incubated with denatured total barley RNA. Then, 30 μl reaction mixtures (1 μg RNA, 1 μM protein in 15 mM Tris-HCl (pH 8.0), 15 mM NaCl, 50 mM KCl, and 2.5 mM EDTA) were incubated at 25°C for 90 min. RNase F1 (Sigma) was included as a positive control. For analysis by the Bioanalyzer 2100 (Agilent Technologies, USA) 10 μl of sample were used.

To test the consumption of native rabbit rRNA, purified AVR_A_ effectors were incubated with 20 μl of rRNA from rabbit reticulocyte lysate (Promega) following the method of Kao et al. 2001 [37]. 30 μl reaction mixtures (20 μl rabbit reticulocyte lysate, 1μM purified AVR_A_ effectors in 15 mM Tris-HCl pH 8.0, 15 mM NaCl, 50 mM KCl, 2.5 mM EDTA) were incubated at 25°C. After 60 or 30 min, the reaction was terminated by adding 20 μl phenol/chloroform and was vortexed for 30 seconds. Samples were sedimented at 14,000 rpm for 15 min and 30 μl of the aqueous layer was removed and mixed with 6 μl electrophoresis loading buffer. For analysis by the Bioanalyzer 2100 (Agilent Technologies, USA) 10 μl of sample were used.

### Yeast two-hybrid assays

*Mla* variants were cloned into the pLexA-GW vector [64] for expression with an N-terminal LexA activation domain under the control of a constitutive ADH1 promoter (BD-MLA). The *AVR*_*a*_ variants were cloned into pB42AD-GW [64] for expression with an N-terminal B42 activation domain followed by the HA tag under the control of an inducible GAL1 promoter (*AD-AVR*_*a*_). Using the lithium acetate method [65], *Mla* bait constructs and *AVR*_*a*_ prey constructs were co-transformed into the yeast strain EGY4.8 p8op-lacZ and successful transformants were selected by colony growth on SD-UHW/Glu (4% (w/v) Glucose, 0.139% (w/v) yeast synthetic drop-out medium pH 5.8 without uracil, histidine, tryptophan, 0.67% (w/v) BD Difco yeast nitrogen base, and 2% (w/v) Bacto Agar). Yeast transformants were grown to OD_600_ 1 in liquid SD-UHW/Glu before harvesting cells for serial dilution on SD-UHW/Gal/Raf media (SD-UHW without glucose but with 2% (w/v) Galactose 1% (w/v) Raffinose, with (-UHW) or without Leucine (-UHWL)) and incubated for 14 days at 30°C.

### Yeast protein extraction

For protein extraction, 10 ml of co-transformed yeast strains were grown to an OD_600_ of 1 in SD-UHW/Gal/Raf liquid medium at 30°C with shaking at 200 rpm. The proteins were precipitated using the ammonium acetate method (modified from Karginov and Agaphonov et al., 2016 [66]). In short, cells were harvested by centrifugation at 700 x*g* for 5 min. The pellets were resuspended in 200 μl NH_4_-acetate buffer (1 M NH_4_(CH_3_COO), 150 mM NaCl, 30 mM Tris-HCl, pH 7.5, 10 mM PMSF, 5 mM EDTA, and one tablet of Protease Inhibitor Cocktail (Roche). The yeast suspension was transferred into BeadBug-prefilled tubes with 0.5-mm silica glass beads (Sigma) and ground in a Precellys homogenizer (two times at 6,200 rpm for 30 sec, break: 15 sec). Afterwards, the DNA was sheared using a Diogenode Bioruptur ultrasonic water bath (twice for 30 sec at high power, break: 90 sec). The suspension without the beads was transferred into a Protein LoBind tube (Eppendorf). The glass beads were washed three times with 250 μl NH_4_-acetate buffer. The washes were combined with the suspension and incubated for 1.5 h on ice. Precipitated proteins were harvested by centrifugation (16,000 x *g* for 10 min). Precipitates were washed with 1 ml 1 M NaCl and the pellet was resuspended with 200 μl Urea-SDS sample buffer (50 mM Tris-HCl, pH 6.8, 2% SDS, 8 M Urea, 1% ß-mercaptoethanol, 2 mM EDTA, 5% glycerol, and 0.004% bromophenol blue) at room temperature. Resuspension in urea-SDS buffer and omission of the boiling step is essential for detection of LexA-MLA fusion proteins. For western blotting, 10–15 μl of the sample were loaded on 8% or 12% SDS page gels, blotted onto PVDF membranes and probed with either anti-HA (Merck, clone 3F10, RRID:AB_390914) or anti-LexA (Santa Cruz, Biotechnology, sc7544, RRID:AB_627883) primary antibodies, followed by incubation with secondary anti-rat (Santa Cruz Biotechnology, sc2065, RRID:AB_631756) or anti-mouse IgG-HRP antibodies (Santa Cruz Biotechnology, sc2005, RRID:AB_631736) for the detection of AVR_A_ or MLA proteins, respectively. HA and LexA fusion proteins were detected by HRP activity on SuperSignal West Femto Maximum Sensitivity Substrate (Thermo Fisher 34095) using a Gel Doc XR and gel documentation system (Bio-Rad).

## Supporting information

S1 Fig Transient co-expression of *AVR*_*a6*_ candidates with *Mla6* in cultivar (*cv*.) Golden Promise barley protoplasts. Transient co-expression of EV or cDNAs of *BLGH_00709* or *BLGH_00697* lacking their respective signal peptides together with *Mla6* and *pUBI*:*Luciferase* in *cv*. Golden Promise protoplasts. The LUC activity relative to the EV sample was measured as a proxy for cell death 16 hours post transfection. Bar diagrams represent mean relative LUC activity of eight transfections, which are represented by dots, while the standard deviation is indicated by error bars. Significant differences between samples were analyzed using a one-way ANOVA and siginificant difference is indicated by different letters. Calculated p-value: *p* = 0.000331
(TIF)

S2 Fig Association of *AVR*_*a6*_ transcriptomic data with phenotypes of *Bgh* isolates on *Mla6* NILs. The table depicts infection phenotypes of 27 *Bgh* isolates on barley *Mla6* near-isogenic lines (NILs) [11,28] of the cultivar (*cv*.) Manchuria and *cv*. Pallas, a heatmap of the fragments per kilobase million (fpkm) expression data of *AVR*_*a6*_, *BLGH_07092* and *AVR*_*a6*_ family members *BLGH_00698*, *BLGH_00697* and *BLGH_00700* and a list of the deduced AVR_A6_ proteins expressed by each isolate.
(TIF)

S3 Fig Schematic illustration of splice site mutations in *AVR*_*a6*,_ which presumably lead to virulence of *Bgh* isolates on *Mla6* NILs. (A) Schematic illustration depicting the three observed splice site mutations: Splice donor site, splice branch point, and splice acceptor site mutations. (B) Schematic illustration of the genomic, transcriptomic, and deduced protein sequences of the three *AVR*_*a6*_ effector variants: *AVR*_*a6*_, *AVR*_*a6*_*-V1*, and *AVR*_*a6*_*-V2*. Depicted is the mutation in the consensus sequence of the branch point in *AVR*_*a6*_*-V1* transcripts of *Bgh* isolates CC66 and CC148 and the splice donor site mutation in *AVR*_*a6*_*-V2*, which can be found in transcripts and the genome of *Bgh* isolate K1. This splice site mutation likely lead to an intron retention, which is supported by RNA-seq reads. Intron retention introduces an early stop codon leading to anticipated truncation of the AVR_A6_-V1 and AVR_A6_-V2 proteins.
(TIF)

S4 Fig Alignment of DNA, RNA, and protein sequences of AVR_A6_, AVR_A6_-V1 and AVR_A6_-V2 variants including the signal peptide. (A) DNA sequence alignment of *AVR*_*a6*_, *AVR*_*a6*_*-V1*, and *AVR*_*a6*_*-V2*. The sequence of *AVR*_*a6*_*-V1* was deduced from RNA-seq reads. (B) RNA sequence alignment of *AVR*_*a6*_, deduced *AVR*_*a6*_*-V1*, and *AVR*_*a6*_*-V2*. (C) Protein sequence of AVR_A6_, deduced AVR_A6_-V1, and AVR_A6_-V2.
(TIF)

S5 Fig Overlay of predicted AVR_A6_ structure (red) with the X-ray crystallography structure of CSEP0064 (yellow) (PDB ID: 6fmb). (TIF)

S6 Fig Examination of the intron characteristic for RALPHs in *Bgt* and *Bgh* avirulence effectors. (A) Position of the intron, which was found to be characteristic for RALPH-like effectors in the gene models of *Bgt* and *Bgh AVR* effectors. Black boxes are the 5′ UTR and 3′ UTR, white boxes are the gene coding regions, dark grey boxes denote the signal peptides, and light grey boxes depict introns. The characteristic intron, which was found in RALPH effectors, is shown in yellow. (B) Protein sequence alignment of *Bgt* and *Bgh* avirulence effectors showing the amino acid similarity and identity using grey and black backgrounds, respectively. Red arrows depict the relative position of the intron. Two black bars at positions 37 and 133 of the alignment show two characteristic cysteines present in all effectors except for AVR_A13_, which are predicted to form a disulfide bond.
(TIF)

S7 Fig DNA sequence alignments of *Bgh* and *Bgt* AVR effectors. (A) DNA sequence alignment of *Bgt* and *Bgh* avirulence effectors including the signal peptide. Yellow background depicts the characteristic intron in RALPH effectors. B) Alignment of the intron sequence, which can be found in *Bgt* and *Bgh* RALPH avirulence effectors. Intron gDNA sequence alignment depicting identical nucleotides with a black background.
(TIF)

S8 Fig Size exclusion chromatography and SDS-PAGE of purified AVR_A6_, AVR_A10_, and AVR_A13_ effector proteins. (A) Size exclusion chromatogram (SEC) of AVR_A6_, AVR_A10_, and AVR_A13_ showing absorbance at 280 nm (y-axis) against the retention volume (ml) (x-axis) and the respective fraction above (A34 and A36 for AVR_A6_, A31 and A33 for AVR_A10_; A32 and A34 for AVR_A13_), which was used for further RNase activity assays. (B) Stain-free SDS-PAGE (Bio-rad) showing the fractions of purified AVR_A6_, AVR_A10_, and AVR_A13_ proteins used for further RNase activity assays with a white arrow. AVR_A_ protein fractions were separated on a 12% polyacrylamide gel and visualized by the ChemiDoc MP Imaging System (170–8280).
(TIF)

S9 Fig Protein quality control of AVR_A6_, AVR_A10_, and AVR_A13_. (A) Size exclusion chromatogram of AVR_A6_, AVR_A10_, and AVR_A13_ purified from insect cells. Fractions eluted at 18.5, 17, 17.5ml of AVR_A6_, AVR_A10_, and AVR_A13_ proteins were verified by SDS-PAGE (indicated by red, green and blue arrows) and used for further RNase activity assays. (B) After size exclusion, insect cell-purified AVR_A6_, AVR_A10_, and AVR_A13_ proteins or T1 RNase were incubated with denatured *Hv*RNA. All samples were separated on non-denaturing 2% agarose gels and analyzed on a Bioanalyzer to determine for RNA degradation.
(TIF)

S10 Fig Amino acid sequence alignment of M61111, M66111, M16666, and M11166. Colored boxes depict different domains of the receptors: blue = CC-domain, green = NB-ARC domain, red = LRR as defined previously in [19]. Grey boxes depict individual LRRs.
(TIF)

S11 Fig *N*. *benthamiana* corresponding to results of Fig 5. Pictures were taken under UV light (302 nm) at 5 days post transformation.
(TIF)

S12 Fig Amino acid sequence alignment of MLA10, MLA22, MLA10LRR22, and MLA22LRR10 receptors. Colored boxes depict different domains of the receptors: blue = CC-domain, green = NB-ARC domain, red = LRR as defined previously in [19]. Grey boxes depict individual LRRs.
(TIF)

S13 Fig *N*. *benthamiana* leaves corresponding to results shown in Fig 8. Pictures were taken at 5 days post transformation.
(TIF)

S14 Fig HR indices of *N*. *benthamiana* leaf infiltrations. (A) HR index used for scoring cell death in *N*. *benthamiana*. 0 = no cell death, 1 = weak chlorosis of infiltrated spot, 2 = chlorosis, 3 = strong chlorosis with rare spots of collapsed, dead leaf material, 4 = strong cell death with collapsed leaf material. The color of the frames around cell death pictures indicates HR indices in stacked bar plots B-E. (B–E) Stacked bar plots showing the count of individual HR indices from independent leaf infiltrations. Significance of cell death scores was calculated by Fisher’s exact test and an asterisk depicts p < 0.05: (B) MLA6, AVR_A6_-1: 8.68xe^-13^; MLA1, AVR_A1_: 2.6xe^-11^ (C) M11166, AVR_A6_-1: 3.98xe^-12^; (D) MLA10, AVR_A10_: 1.25xe^-10^; MLA22LRR10, AVR_A10_: 3.07xe^-07^; MLA22, AVR_A22_: 7.74xe^-08^; MLA10LRR22, AVR_A22_: 5.8xe^-07^ (E) MLA10, AVR_A10_: 3.07xe^-29^; MLA10, chimera26: 1.54xe^-13^; MLA10, chimera29: 3.73xe^-12^; MLA22, AVR_A22_: 2.77xe^-49^; MLA22, chimera14: 6.68xe^-28^; MLA22, chimera22: 6.39xe^-14^; MLA22, chimera24: 1.5xe^-11^, MLA22; chimera26: 9.59xe^-09^.
(TIF)

S1 Table Top-ranking *AVR*_*a6*_ candidates in the transcriptome-wide association study (TWAS) determined by gene-wise calling. * *The table columns show the new and former BLGH_ID, the description, the scaffold localization and the *p*-value of the top-ranking candidates for the gene-wise association of *Bgh* transcriptomes with infection phenotypes on *Mla6* near-isogenic lines (NILs). Color codes depict top-ranking *AVR*_*a6*_ candidates and are consistent with the color code used in Fig 1A: bright green: CSEP0254 paralogues, dark green: BLGH_07092, dark red: BLGH_00697, bright red: BLGH_00700.
(TIF)

S2 Table Top-ranking *AVR*_*a6*_ candidates in the transcriptome-wide association study (TWAS) determined by variant-wise calling. * *The table columns depict the scaffold localization, the effect that the mutation has on the reference gene (non-synonymous mutation, gained stop codon), the codon change and the respective aa exchange, a gene description, the CSEP_ID and the *p*-value of the top-ranking candidates for the variant-wise association of *Bgh* transcriptomes with infection phenotypes on *Mla6* near-isogenic lines (NILs). Color codes designate top-ranking *AVR*_*a6*_ candidates and are consistent with the color code used in Fig 1A: bright green: CSEP0254 paralogues, dark green: BLGH_07092 and bright red: BLGH_00700.
(TIF)

S3 Table Primers used in this study. (TIF)
