# Supplementary figures and images for: The leucine-rich repeats in allelic barley MLA immune receptors define specificity towards sequence-unrelated powdery mildew avirulence effectors with a predicted common RNase-like fold
Source: PLoS Pathog. 2021 Feb 3;17(2):e1009223. doi: 10.1371/journal.ppat.1009223 (PMC7857584; doi:10.1371/journal.ppat.1009223)

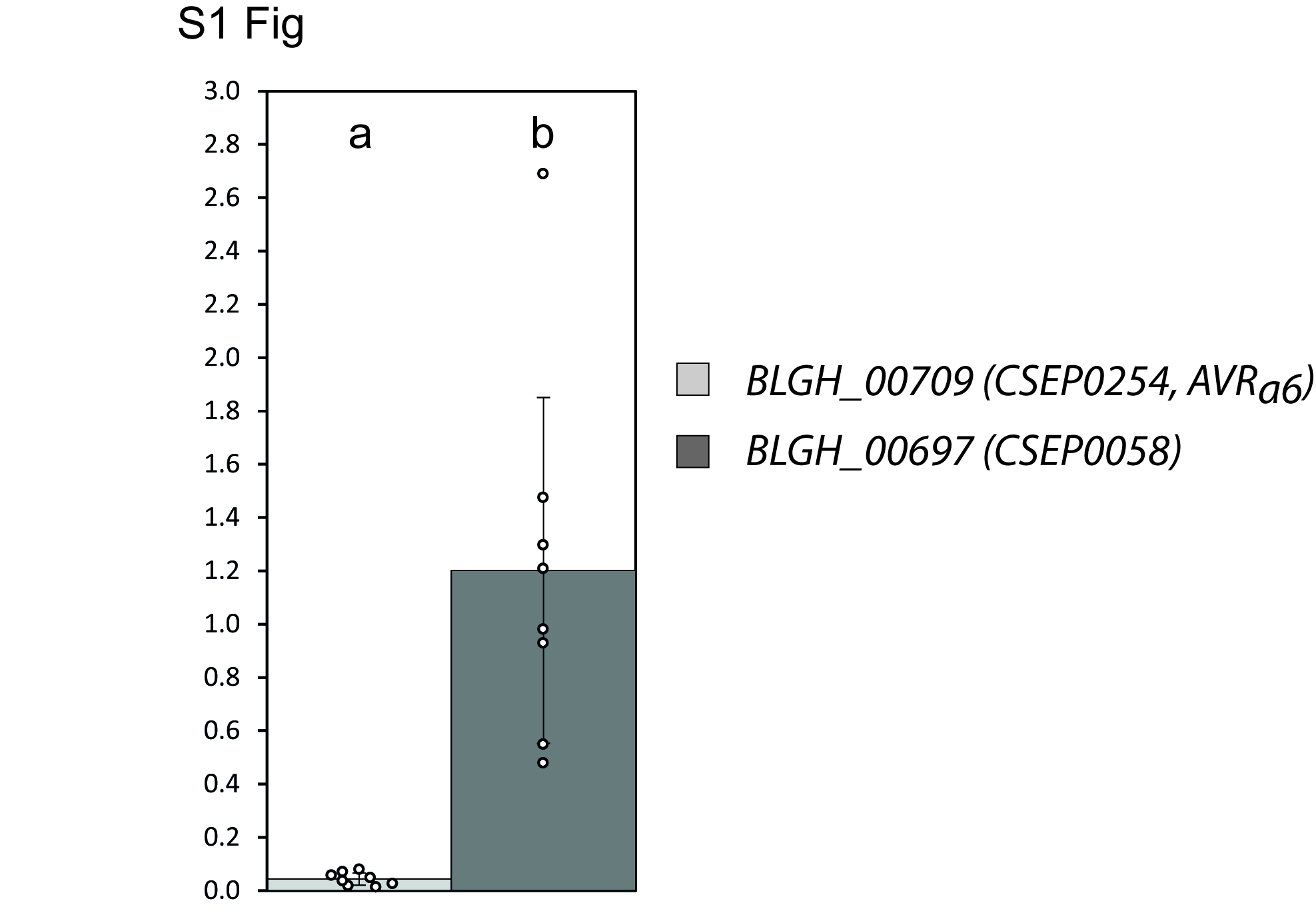

Supplement: S1 Fig — Transient co-expression of EV or cDNAs of BLGH_00709 or BLGH_00697 lacking their respective signal peptides together with Mla6 and pUBI:Luciferase in cv. Golden Promise protoplasts. The LUC activity relative to the EV sample was measured as a proxy for cell death 16 hours post transfection. Bar diagrams represent mean relative LUC activity of eight transfections, which are represented by dots, while the standard deviation is indicated by error bars. Significant differences between samples were analyzed using a one-way ANOVA and siginificant difference is indicated by different letters. Calculated p-value: p = 0.000331 (TIF) [file ppat.1009223.s001.tif]

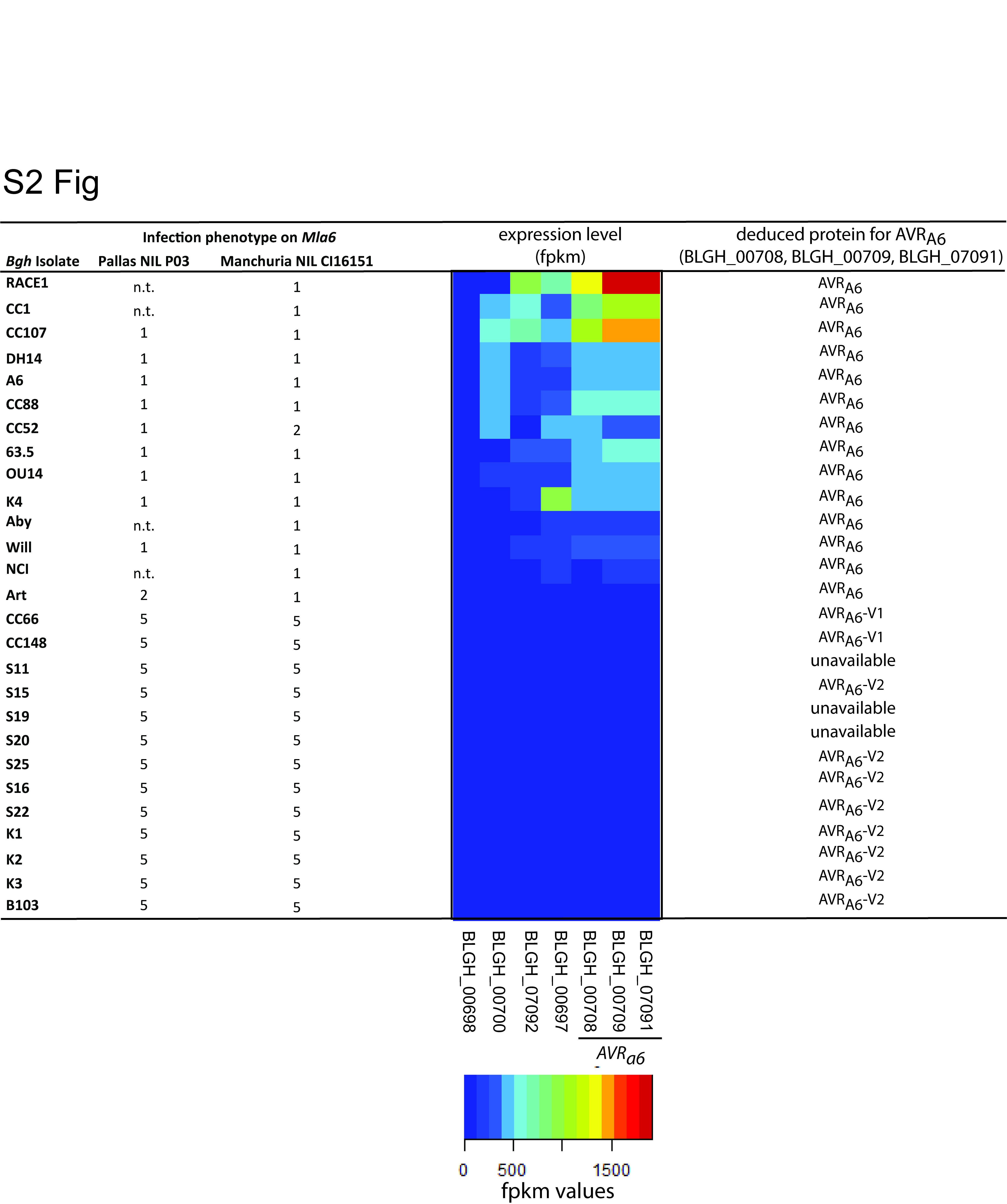

Supplement: S2 Fig — The table depicts infection phenotypes of 27 Bgh isolates on barley Mla6 near-isogenic lines (NILs) [11,28] of the cultivar (cv.) Manchuria and cv. Pallas, a heatmap of the fragments per kilobase million (fpkm) expression data of AVRa6, BLGH_07092 and AVRa6 family members BLGH_00698, BLGH_00697 and BLGH_00700 and a list of the deduced AVRA6 proteins expressed by each isolate. (TIF) [file ppat.1009223.s002.tif]

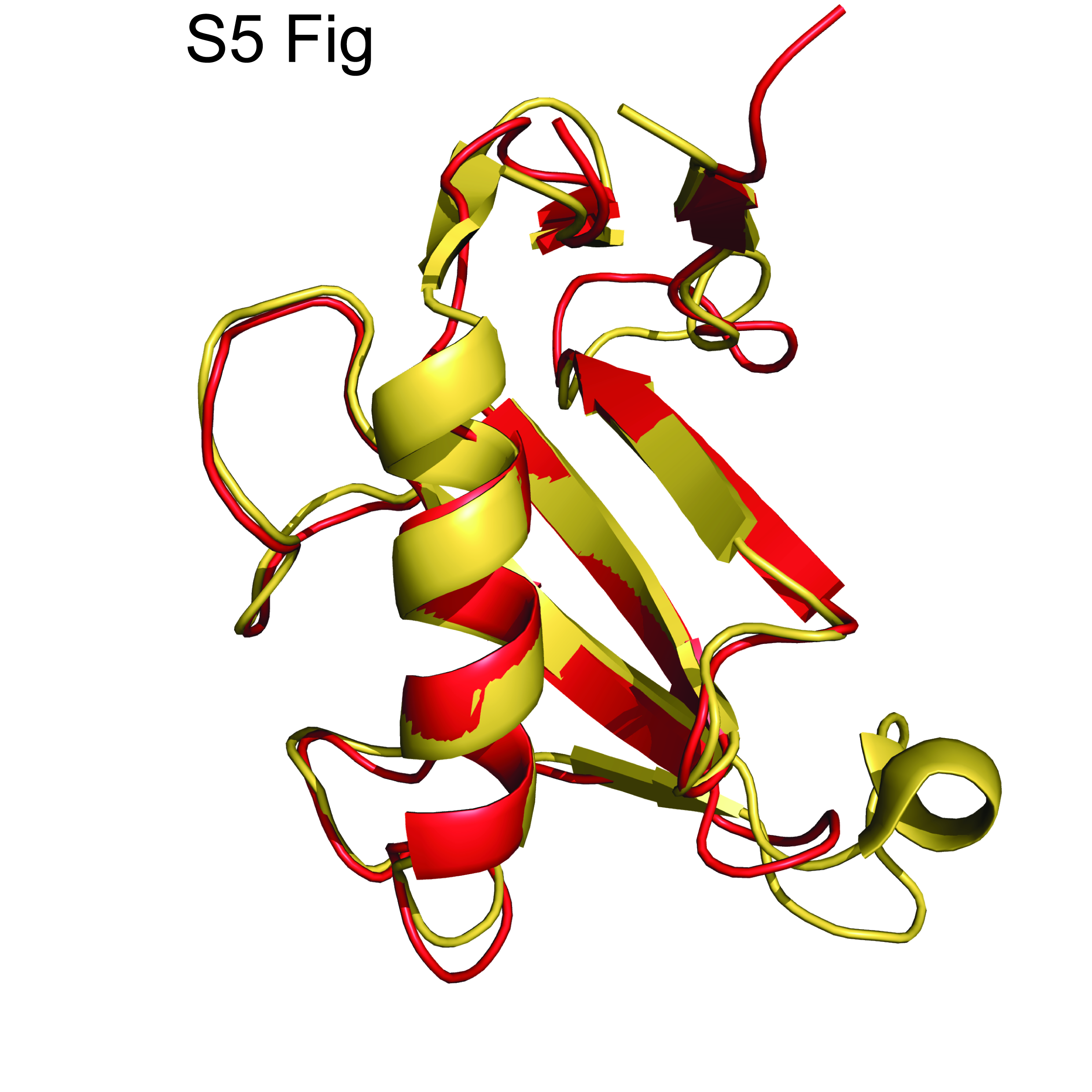

Supplement: S5 Fig — (TIF) [file ppat.1009223.s005.tif]

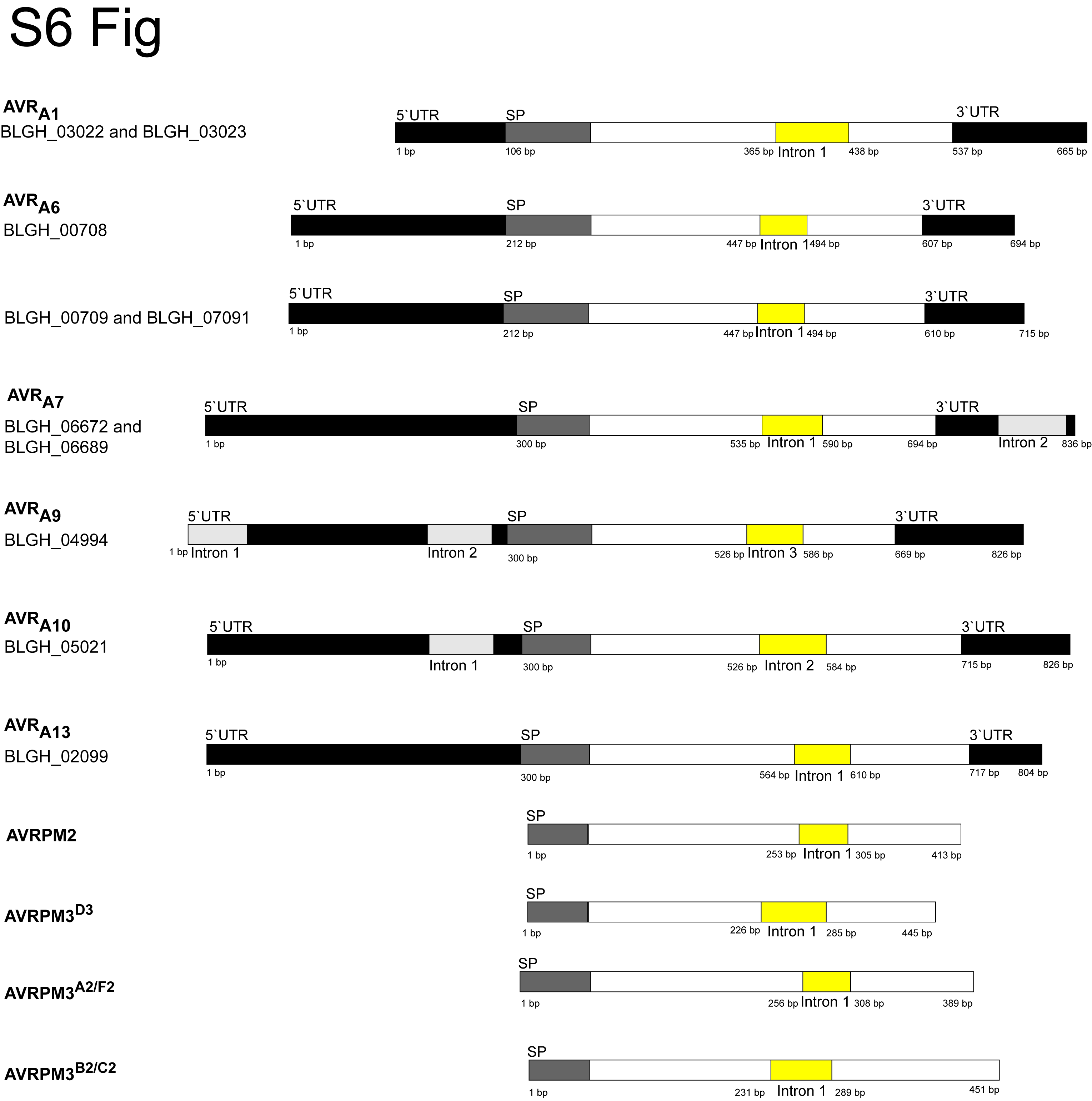

Supplement: S6 Fig — (A) Position of the intron, which was found to be characteristic for RALPH-like effectors in the gene models of Bgt and Bgh AVR effectors. Black boxes are the 5′ UTR and 3′ UTR, white boxes are the gene coding regions, dark grey boxes denote the signal peptides, and light grey boxes depict introns. The characteristic intron, which was found in RALPH effectors, is shown in yellow. (B) Protein sequence alignment of Bgt and Bgh avirulence effectors showing the amino acid similarity and identity using grey and black backgrounds, respectively. Red arrows depict the relative position of the intron. Two black bars at positions 37 and 133 of the alignment show two characteristic cysteines present in all effectors except for AVRA13, which are predicted to form a disulfide bond. (TIF) [file ppat.1009223.s006.tif]

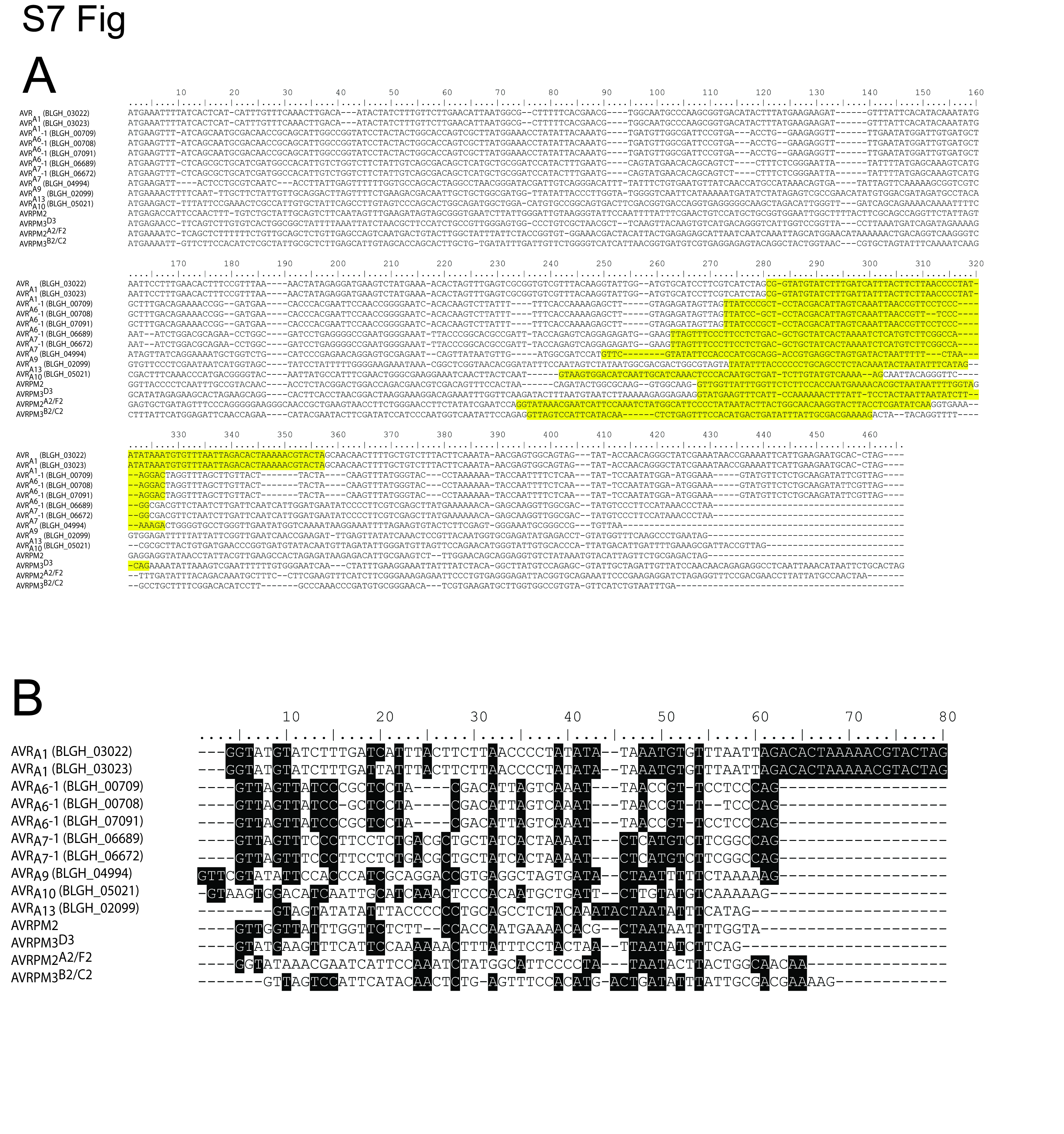

Supplement: S7 Fig — (A) DNA sequence alignment of Bgt and Bgh avirulence effectors including the signal peptide. Yellow background depicts the characteristic intron in RALPH effectors. B) Alignment of the intron sequence, which can be found in Bgt and Bgh RALPH avirulence effectors. Intron gDNA sequence alignment depicting identical nucleotides with a black background. (TIF) [file ppat.1009223.s007.tif]

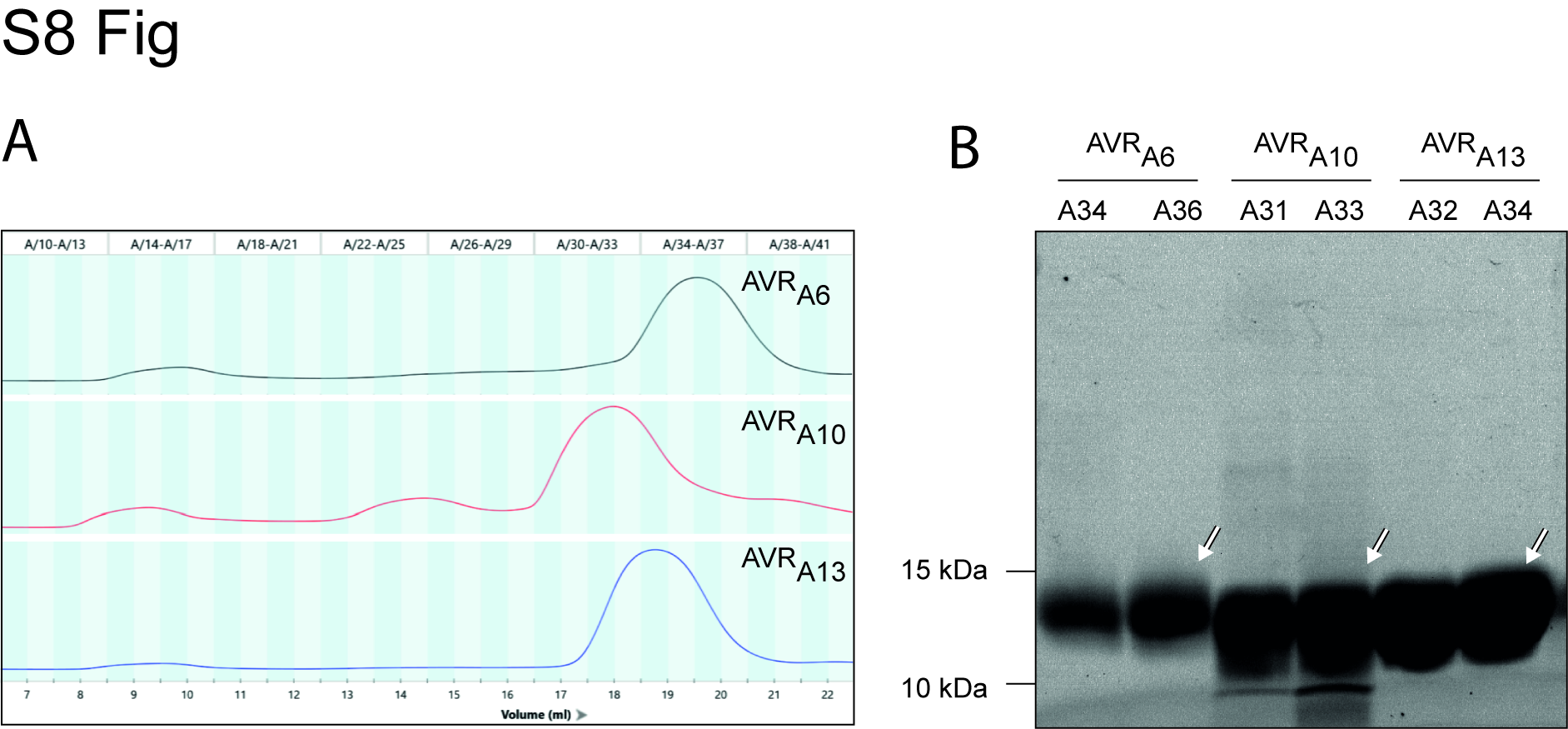

Supplement: S8 Fig — (A) Size exclusion chromatogram (SEC) of AVRA6, AVRA10, and AVRA13 showing absorbance at 280 nm (y-axis) against the retention volume (ml) (x-axis) and the respective fraction above (A34 and A36 for AVRA6, A31 and A33 for AVRA10; A32 and A34 for AVRA13), which was used for further RNase activity assays. (B) Stain-free SDS-PAGE (Bio-rad) showing the fractions of purified AVRA6, AVRA10, and AVRA13 proteins used for further RNase activity assays with a white arrow. AVRA protein fractions were separated on a 12% polyacrylamide gel and visualized by the ChemiDoc MP Imaging System (170–8280). (TIF) [file ppat.1009223.s008.tif]

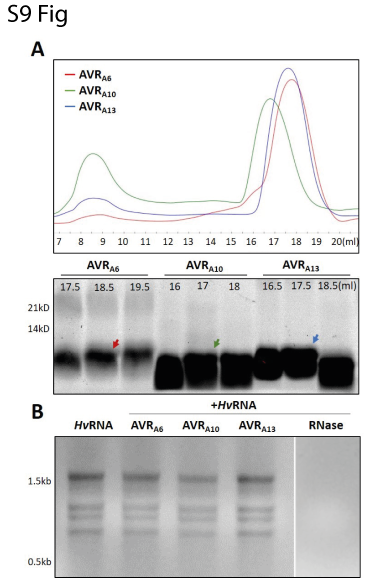

Supplement: S9 Fig — (A) Size exclusion chromatogram of AVRA6, AVRA10, and AVRA13 purified from insect cells. Fractions eluted at 18.5, 17, 17.5ml of AVRA6, AVRA10, and AVRA13 proteins were verified by SDS-PAGE (indicated by red, green and blue arrows) and used for further RNase activity assays. (B) After size exclusion, insect cell-purified AVRA6, AVRA10, and AVRA13 proteins or T1 RNase were incubated with denatured HvRNA. All samples were separated on non-denaturing 2% agarose gels and analyzed on a Bioanalyzer to determine for RNA degradation. (TIF) [file ppat.1009223.s009.tif]

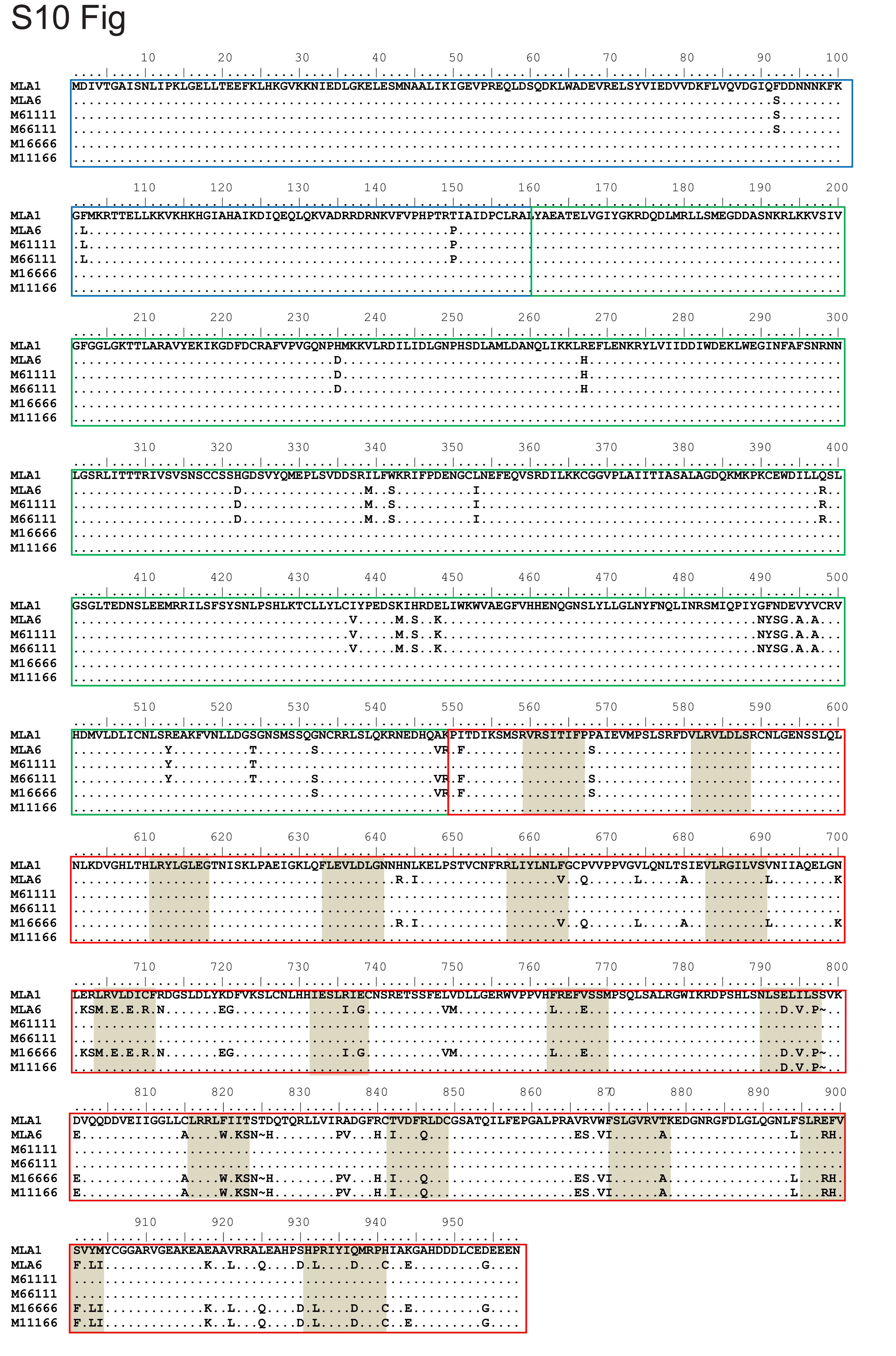

Supplement: S10 Fig — Colored boxes depict different domains of the receptors: blue = CC-domain, green = NB-ARC domain, red = LRR as defined previously in [19]. Grey boxes depict individual LRRs. (TIF) [file ppat.1009223.s010.tif]

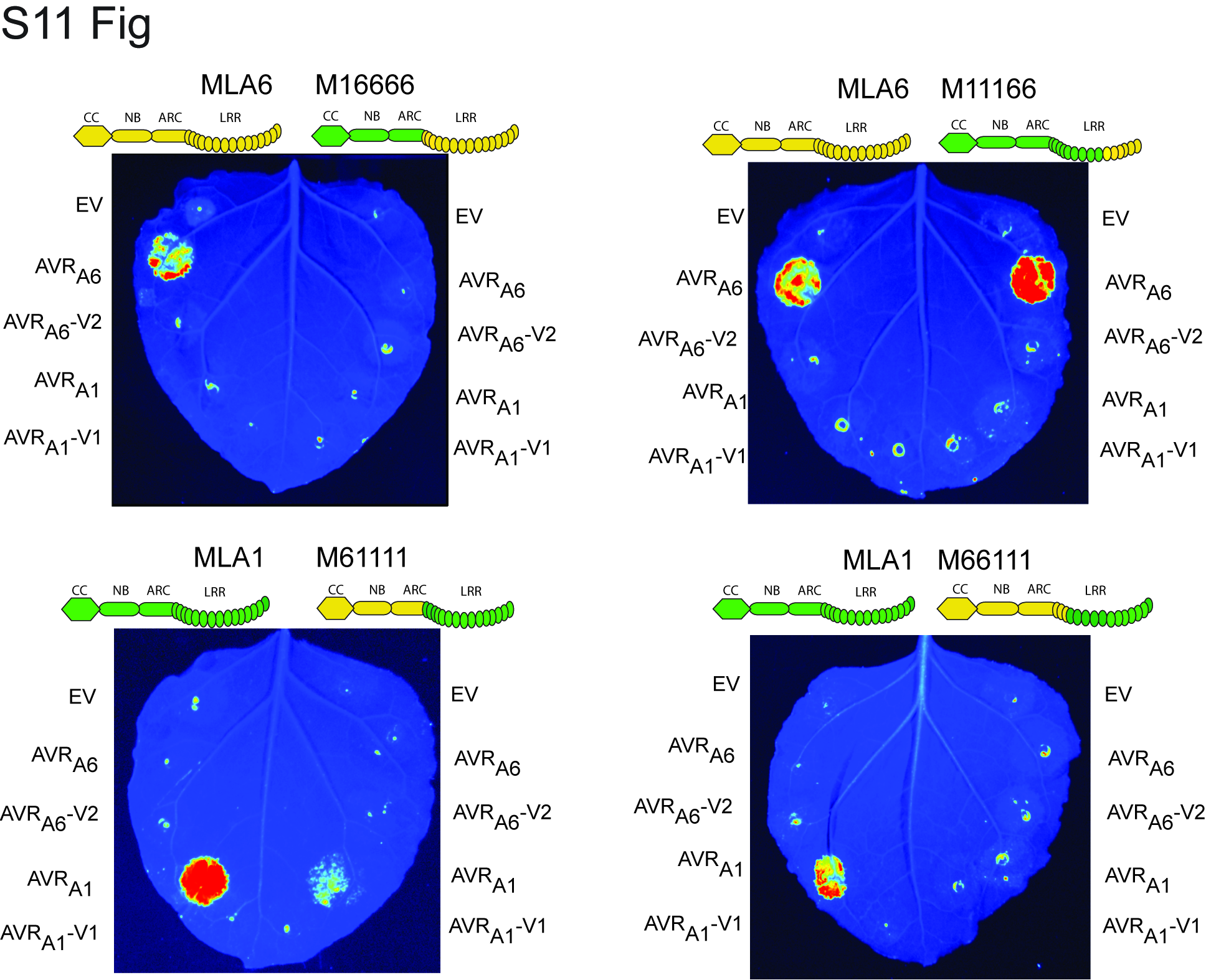

Supplement: S11 Fig — Pictures were taken under UV light (302 nm) at 5 days post transformation. (TIF) [file ppat.1009223.s011.tif]

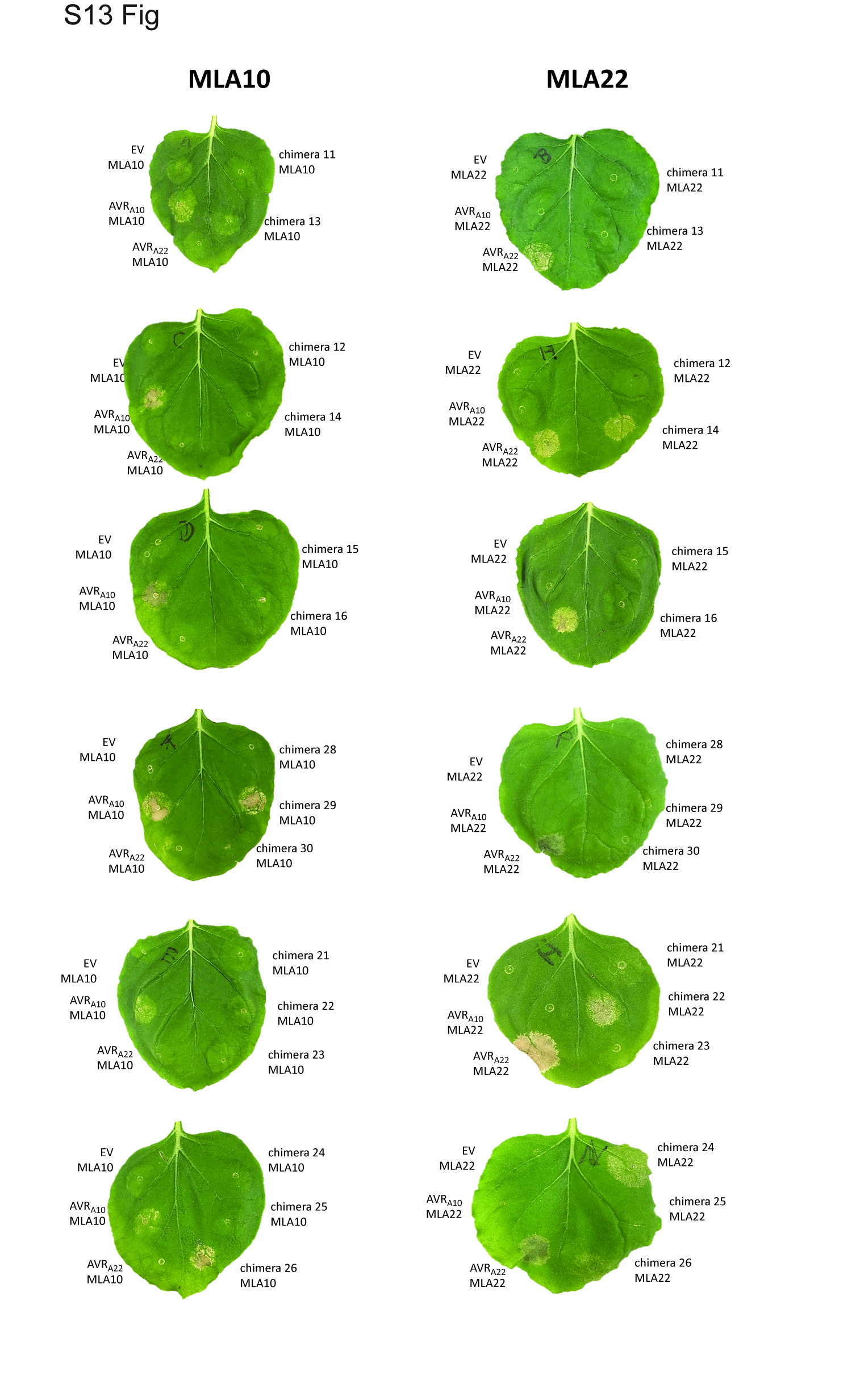

Supplement: S13 Fig — Pictures were taken at 5 days post transformation. (TIF) [file ppat.1009223.s013.tif]

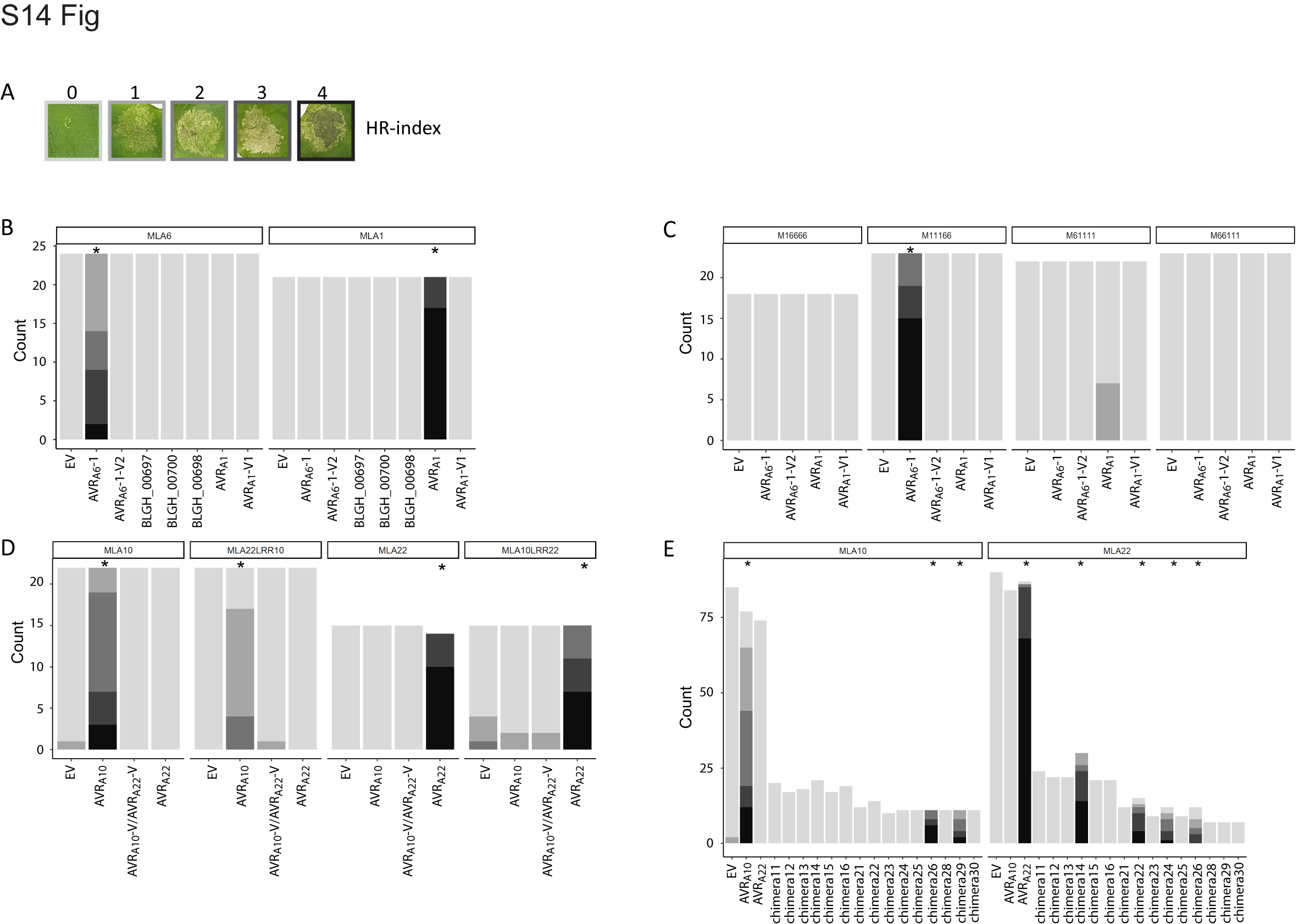

Supplement: S14 Fig — (A) HR index used for scoring cell death in N. benthamiana. 0 = no cell death, 1 = weak chlorosis of infiltrated spot, 2 = chlorosis, 3 = strong chlorosis with rare spots of collapsed, dead leaf material, 4 = strong cell death with collapsed leaf material. The color of the frames around cell death pictures indicates HR indices in stacked bar plots B-E. (B–E) Stacked bar plots showing the count of individual HR indices from independent leaf infiltrations. Significance of cell death scores was calculated by Fisher’s exact test and an asterisk depicts p < 0.05: (B) MLA6, AVRA6-1: 8.68xe-13; MLA1, AVRA1: 2.6xe-11 (C) M11166, AVRA6-1: 3.98xe-12; (D) MLA10, AVRA10: 1.25xe-10; MLA22LRR10, AVRA10: 3.07xe-07; MLA22, AVRA22: 7.74xe-08; MLA10LRR22, AVRA22: 5.8xe-07 (E) MLA10, AVRA10: 3.07xe-29; MLA10, chimera26: 1.54xe-13; MLA10, chimera29: 3.73xe-12; MLA22, AVRA22: 2.77xe-49; MLA22, chimera14: 6.68xe-28; MLA22, chimera22: 6.39xe-14; MLA22, chimera24: 1.5xe-11, MLA22; chimera26: 9.59xe-09. (TIF) [file ppat.1009223.s014.tif]

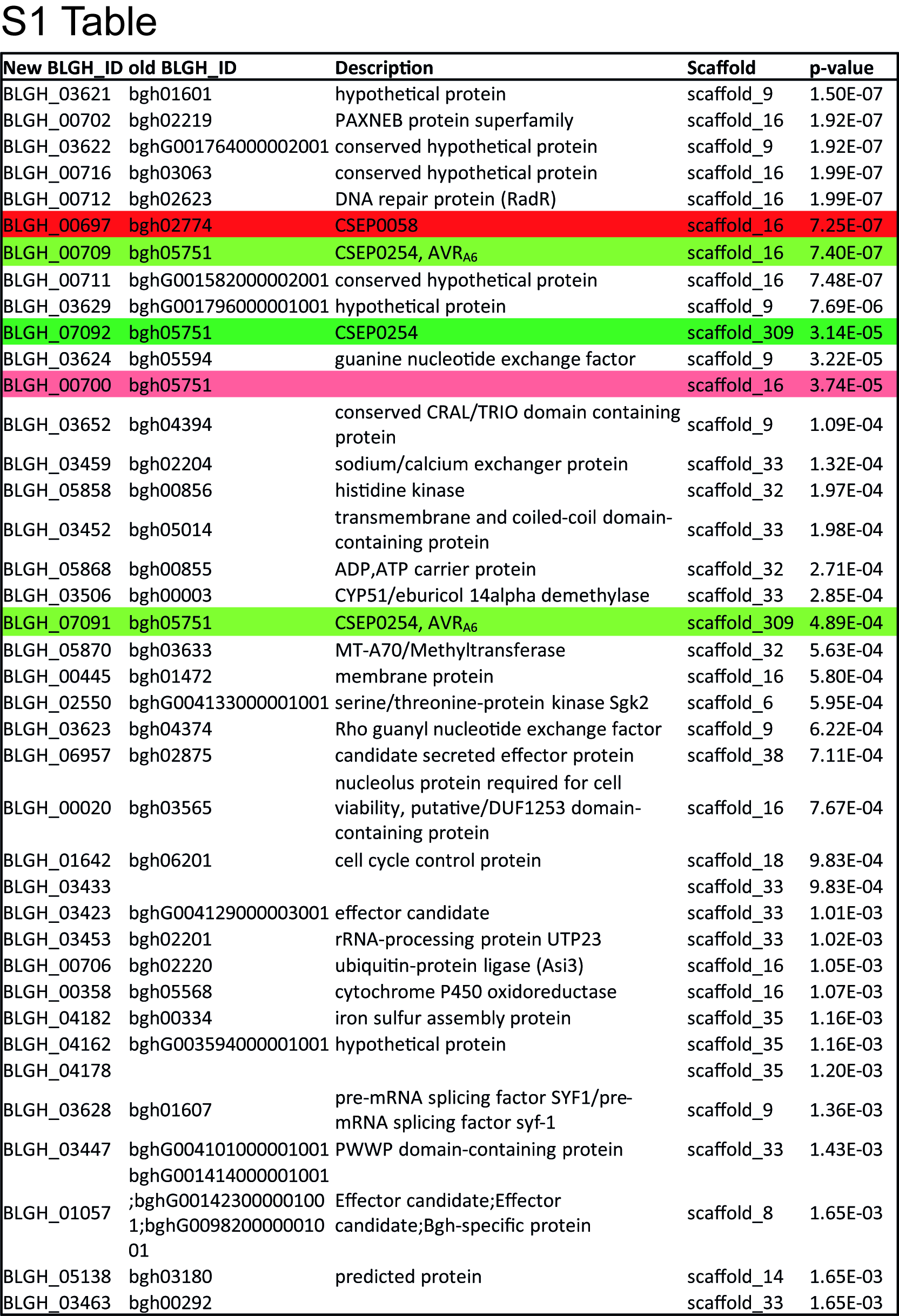

Supplement: S1 Table — * *The table columns show the new and former BLGH_ID, the description, the scaffold localization and the p-value of the top-ranking candidates for the gene-wise association of Bgh transcriptomes with infection phenotypes on Mla6 near-isogenic lines (NILs). Color codes depict top-ranking AVRa6 candidates and are consistent with the color code used in Fig 1A: bright green: CSEP0254 paralogues, dark green: BLGH_07092, dark red: BLGH_00697, bright red: BLGH_00700. (TIF) [file ppat.1009223.s015.tif]

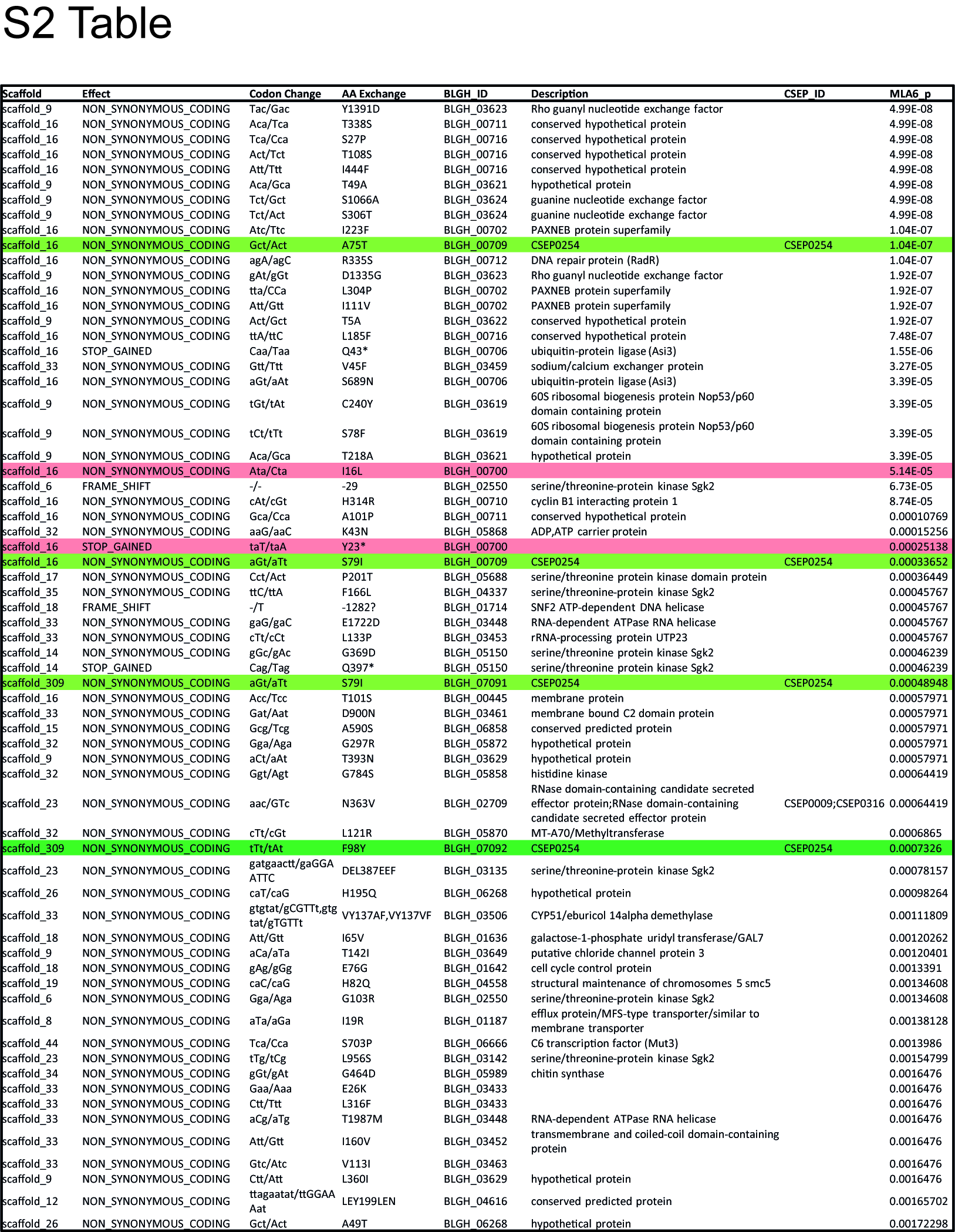

Supplement: S2 Table — * *The table columns depict the scaffold localization, the effect that the mutation has on the reference gene (non-synonymous mutation, gained stop codon), the codon change and the respective aa exchange, a gene description, the CSEP_ID and the p-value of the top-ranking candidates for the variant-wise association of Bgh transcriptomes with infection phenotypes on Mla6 near-isogenic lines (NILs). Color codes designate top-ranking AVRa6 candidates and are consistent with the color code used in Fig 1A: bright green: CSEP0254 paralogues, dark green: BLGH_07092 and bright red: BLGH_00700. (TIF) [file ppat.1009223.s016.tif]

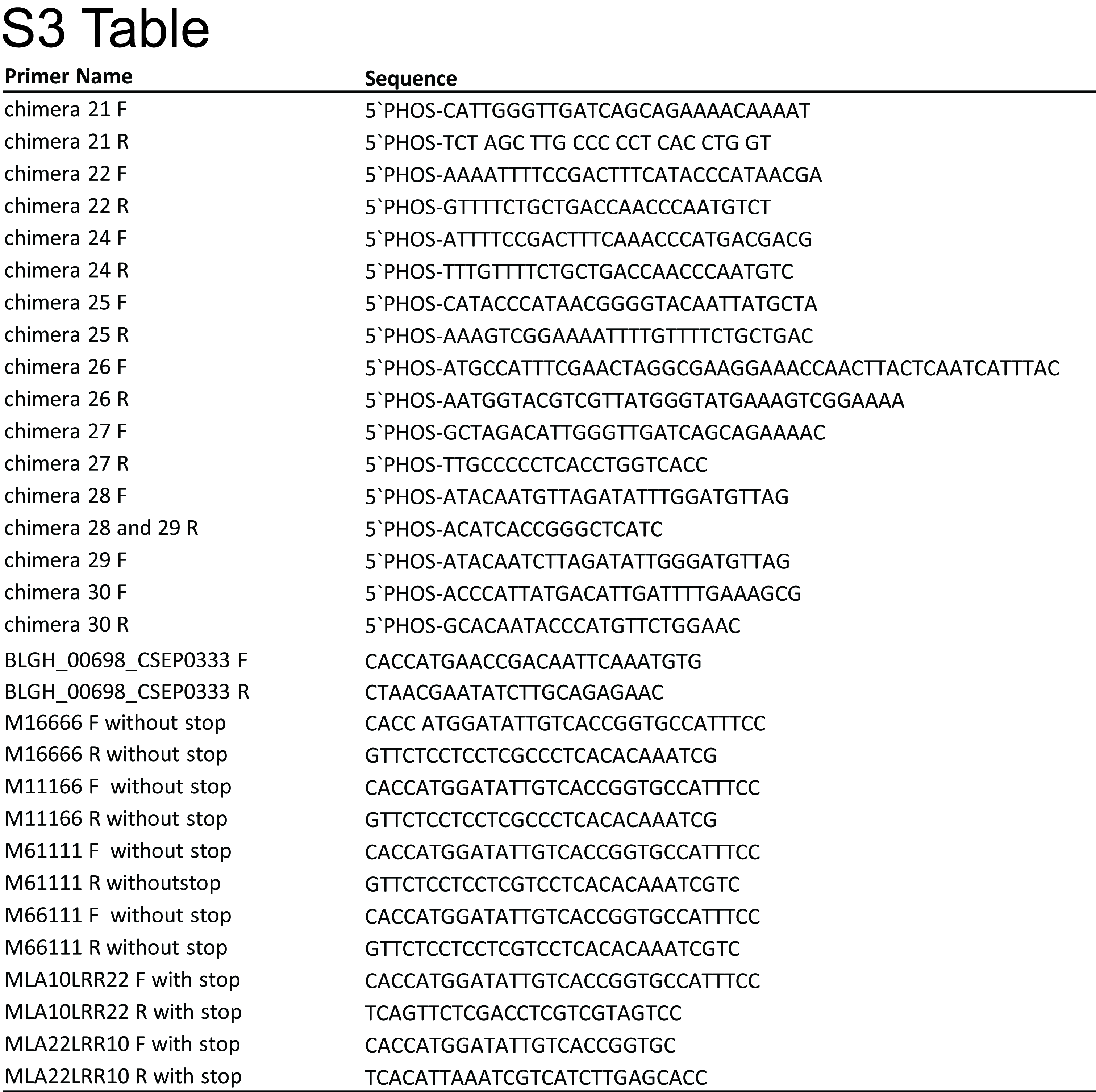

Supplement: S3 Table — (TIF) [file ppat.1009223.s017.tif]
